# NGR‐Modified CAF‐Derived exos Targeting Tumor Vasculature to Induce Ferroptosis and Overcome Chemoresistance in Osteosarcoma

**DOI:** 10.1002/advs.202410918

**Published:** 2025-01-31

**Authors:** Jianxin Du, Xiangwei Meng, Minghao Yang, Guancheng Chen, Jigang Li, Zengjun Zhu, Xuanxuan Wu, Wei Hu, Maojin Tian, Tao Li, Shuai Ren, Peiqing Zhao

**Affiliations:** ^1^ Center of Translational Medicine Zibo Central Hospital Affiliated to Binzhou Medical University Zibo 255036 China; ^2^ Department of Radiology Yantai Affiliated Hospital of Binzhou Medical University Yantai 264100 China; ^3^ State Key Laboratory of Reproductive Medicine and Offspring Health Nanjing Medical University Nanjing 211166 China; ^4^ Department of Orthopedics Zibo Central Hospital Affiliated to Binzhou Medical University Zibo 255036 China; ^5^ School of Medical Laboratory Shandong Second Medical University Weifang 261042 China; ^6^ Department of Orthopedics Nanjing Jiangbei Hospital Nanjing 210044 China

**Keywords:** cancer‐associated fibroblasts, circ_0004872‐109aa, circ_0004872, NGR, NGR‐109aa‐Exos, Osteosarcoma

## Abstract

Osteosarcoma (OS) chemoresistance presents a significant clinical challenge. This study aims to investigate the potential of using tumor vascular‐targeting peptide NGR‐modified cancer‐associated fibroblasts (CAFs)‐derived exosomes (exos) to deliver circ_0004872‐encoded small peptides promoting autophagy‐dependent ferroptosis to reverse chemoresistance in OS. Through combined single‐cell transcriptome analysis and high‐throughput sequencing, it identified circ_0004872 associated with chemoresistance. Subsequent experiments demonstrated that the small peptide encoded by this Circular RNA (circRNA) can effectively reverse chemoresistance by enhancing OS cell sensitivity to chemotherapy via the mechanism of promoting autophagy‐dependent ferroptosis. Moreover, in vitro and in vivo results confirmed the efficient delivery of NGR‐modified CAFs‐derived exo‐packaged circ_0004872‐109aa to tumor cells, thereby improving targeted therapy efficacy. This study not only offers a novel strategy to overcome chemoresistance in OS but also highlights the potential application value of utilizing exos for drug delivery.

## Graphical abstract

This study aims to explore the feasibility of employing NGR‐modified exosomes derived from cancer‐associated fibroblasts to target tumor vasculature, delivering small peptides encoded by circ_0004872 that promote autophagy‐dependent ferroptosis, thereby reversing chemoresistance in osteosarcoma. This research not only presents a promising strategy to overcome chemoresistance in osteosarcoma but highlights the significant potential of exosome‐based drug delivery systems in cancer therapy.

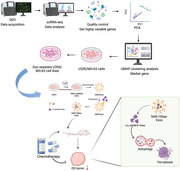

## 1 Introduction

Osteosarcoma (OS) is a highly aggressive and challenging malignant bone tumor that primarily affects children and adolescents.^[^ ^1^, ^2^ ^]^ Despite advances in surgical and chemotherapy regimens that have improved OS cure rates in recent years, chemotherapy resistance remains a major obstacle, adversely affecting treatment outcomes and patient prognosis.^[^ ^3^, ^4^ ^]^ The high recurrence and metastatic rates of OS further complicate and intensify the challenges in treatment.^[^ ^5^ ^]^ Therefore, there is an urgent need to develop new treatment strategies to address chemotherapy resistance in OS and enhance patients' clinical outcomes.^[^ ^6^ ^]^ With the advancement of molecular biology techniques in recent years, researchers have gradually recognized the significant role of the tumor microenvironment in the initiation and progression of tumors, including processes such as intercellular communication, extracellular matrix, and angiogenesis.^[^ ^7^ ^]^ Additionally, our studies on the role of the bone tumor microenvironment in tumorigenesis and progression have made significant advances, particularly in regulating cell‐cell communication and chemotherapy resistance.^[^ ^8^, ^9^ ^]^ These findings provide new perspectives for the development of therapeutic targets for tumors.

Extracellular vesicles (EVs) are nanosized particles secreted by cells that mediate the transfer of bioactive molecules, such as proteins and nucleic acids, thus regulating intercellular communication and biological behaviors.^[^ ^10^, ^11^ ^]^ Emerging evidence highlights the role of EVs in the tumor microenvironment, with studies suggesting that they promote tumor growth and progression through diverse mechanisms.^[^ ^12^, ^13^, ^14^ ^]^ Similarly, circular RNA (circRNA), a class of non‐coding RNA characterized by their stable circular structure, have gained significant attention in tumor research due to their multifaceted functions.^[^ ^15^, ^16^ ^]^ CircRNAs exhibit diverse mechanisms in tumors, including gene expression regulation, protein interactions, and encoding small peptides.^[^ ^17^ ^]^ Among these, circRNA‐encoded small peptides play a crucial role in regulating tumor cell proliferation, migration, and chemotherapy resistance.^[^ ^18^, ^19^, ^20^ ^]^ However, the specific role and mechanism of circRNA and its encoded small peptides in OS resistance remain unclear.^[^ ^21^ ^]^

Tumor vasculature‐targeting peptide NGR is a peptide that can specifically identify and bind to tumor blood vessels, widely used in tumor‐targeted therapy research. The specific binding ability of NGR makes it an ideal carrier for delivering anti‐tumor drugs and gene therapy molecules, enhancing treatment specificity and efficacy.^[^ ^22^ ^]^ Cancer‐associated fibroblasts (CAFs) are a significant component of the tumor microenvironment, contributing to tumor progression and resistance formation through the secretion of EVs. Studies have shown that CAF‐secreted EVs have a notable advantage in delivering therapeutic molecules between tumor cells.^[^ ^23^, ^24^, ^25^ ^]^ Therefore, utilizing NGR‐modified CAFs' EVs for delivering therapeutic molecules can improve treatment specificity and effectiveness.^[^ ^26^ ^]^ Although this strategy theoretically offers significant advantages, its practical application effects and mechanisms require further validation and exploration.^[^ ^27^, ^28^ ^]^

Autophagy‐dependent ferroptosis is a form of cell death caused by the accumulation of iron‐dependent lipid peroxides, which has gradually become a hot topic in anti‐tumor research.^[^ ^29^, ^30^ ^]^ Ferroptosis is not only closely related to the proliferation and migration of tumor cells but also plays a crucial role in regulating the chemotherapy sensitivity of tumor cells.^[^ ^31^ ^]^ Studies have shown that autophagy plays a key regulatory role in the process of ferroptosis by controlling intracellular iron ion levels and lipid metabolism to promote the occurrence of ferroptosis.^[^ ^32^, ^33^, ^34^ ^]^ Therefore, strategies that promote autophagy‐dependent ferroptosis are expected to become a new approach to reversing tumor chemotherapy resistance.^[^ ^35^ ^]^ The high invasiveness and drug resistance of OS cells make exploring the mechanism of autophagy‐dependent ferroptosis particularly important.

Despite previous studies utilizing single‐cell transcriptome sequencing to reveal the complexity of the OS tumor microenvironment, identifying nine major cell types, including osteoblasts,^[^ ^36^ ^]^ the specific mechanisms underlying the interactions between CAFs and malignant cells in the formation of chemoresistance remain poorly understood. This study aims to investigate the mechanism by which NGR‐modified CAF‐derived exosomes (exos) deliver circ_0004872‐encoded small peptides to promote autophagy‐dependent ferroptosis, thereby reversing chemoresistance in OS. Through integrated single‐cell transcriptome analysis, we identified the interactions between CAFs and malignant cells and screened for circRNAs associated with OS chemoresistance. High‐throughput sequencing provided sequencing data from resistant and non‐resistant OS cell lines, and Tagged RNA Affinity Purification (TRAP) technology was used to obtain circRNA‐encoded small peptides.

In vitro experiments involved constructing OS cell models with circRNA knockdown and overexpression to analyze their effects on OS chemoresistance. Concurrently, CAFs and their exos were isolated, and tumor vasculature‐targeting peptide NGR‐modified CAF exos encapsulating circ_0004872‐109aa were created using plasmid transfection and electroporation techniques to verify their efficacy in both in vitro and in vivo experiments. This study will reveal the role of circ_0004872 and its encoded small peptides in OS chemoresistance mechanisms, providing novel therapeutic strategies and theoretical support for clinical applications. This research not only advances the understanding of the molecular mechanisms underlying circRNA involvement in tumor chemoresistance but also broadens insights into intercellular interactions within the tumor microenvironment, offering novel directions for the development of anti‐cancer drugs and therapeutic strategies. Ultimately, the findings of this study will make significant contributions to improving the treatment outcomes and prognosis for OS patients.

## 2 Results

### 2.1 Identification of Cell Types in the Tumor Tissue of OS Patients, Primarily Comprising Osteoblasts

OS, also known as osteogenic sarcoma, is a malignant bone tumor that commonly occurs in adolescents and children under the age of 20. Due to its highly invasive and metastatic nature, it has long posed a challenge in cancer treatment. Therefore, understanding the mechanisms underlying OS development is crucial for improving patient outcomes.^[^ ^37^, ^38^ ^]^ Alterations in the tumor microenvironment play a pivotal role in the malignant progression of OS.^[^ ^39^, ^40^ ^]^

**Figure 1:**
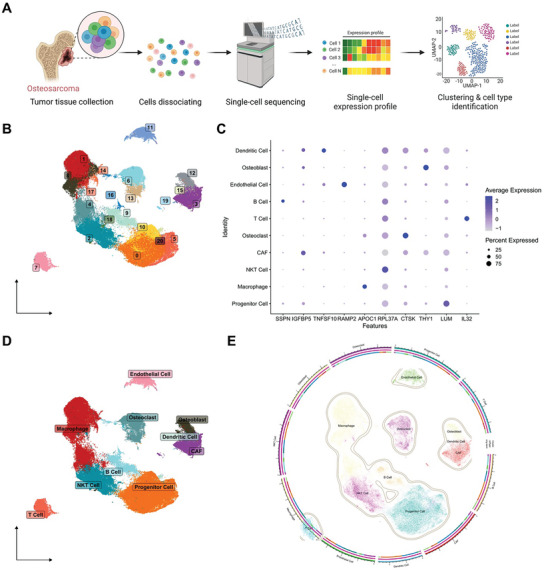
scRNA‐seq Data Cell Clustering and Annotation. Note: A) Schematic diagram illustrating single‐cell data sample acquisition and analysis workflow (Created by Biorender); B) Visualization of UMAP clustering results showing the cell aggregation and distribution in OS samples (N = 11), where each color represents a cluster; C) Expression patterns of known lineage‐specific marker genes in different clusters of OS samples (N = 11), with darker blue indicating higher average expression levels and larger circles representing more cells expressing the gene; D) Visualization of cell annotation results based on UMAP clustering in OS samples (N = 11), where each color represents a cell subpopulation; E) Circular representation of UMAP clustering results demonstrating the cell aggregation and distribution in OS samples (N = 11).

To gain deeper insights into the development of OS and changes within its immune microenvironment, we obtained single‐cell transcriptome sequencing data from the GSE152048 dataset in the GEO database, which includes 11 OS tumor tissue samples (**Figure** **1**A). After integration, filtering, and quality control of the data, the results indicated that PC_1 through PC_20 effectively capture the information contained within the selected highly variable genes, demonstrating significant analytical value (Figure S5, Supporting Information).

Subsequently, we utilized the UMAP algorithm to perform non‐linear dimensionality reduction on the first 20 PCs and conducted clustering analysis with a resolution of 0.5 (Figure S6, Supporting Information). The clustering analysis revealed the presence of 21 clusters, and the expression of marker genes for each cluster was determined (Figure 1B). To annotate the cells, we identified lineage‐specific marker genes by consulting relevant literature and utilized the online platform CellMarker for annotation purposes (Figure 1C). In total, 10 cell types were identified: B Cell, CAF, Dendritic Cell, Endothelial Cell, Macrophage, Natural Killer T (NKT) Cell, Osteoclast, Osteoblast, Progenitor Cell, and T Cell (Figure 1D,E).

In summary, the results demonstrate that the tumor tissue of OS patients predominantly comprises 10 cell types, including Osteoblasts.

### 2.2 Strongest Signaling Between CAF and Malignant Cells in OS Samples

**Figure 2:**
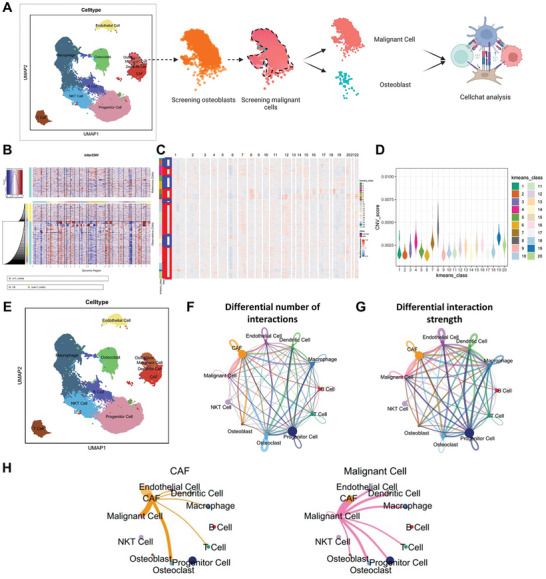
Malignant Cell Extraction and Cell Communication Analysis Results. Note: A) Schematic diagram of the analysis for selecting malignant cells and cell communication analysis with scRNA‐seq data. B) CNV analysis of scRNA‐seq sequencing data, with T cells as reference and observed T cells shown in yellow, and OB in cyan. Blue in the map indicates DNA copy loss, while red indicates DNA copy gain. C) K‐means clustering algorithm clustering the cells from the “observation” in Figure B. D) CNV scores after K‐means clustering. E) UMAP clustering map of scRNA‐seq data after malignant cell extraction. F,G) Circle diagrams showing differences in cell communication in OS samples (N = 11), where the thickness of the lines in Figure F represents the number of pathways, and in Figure G represents the interaction strength. H) Diagram illustrating the differences in cell communication between CAF and malignant cells in OS samples (N = 11), where the line thickness represents the interaction strength.

OS typically originates from osteoblasts or their precursors, with a high tendency for metastasis.^[^ ^41^ ^]^ In order to investigate the variations of these cell types in OS samples, we utilized the “inferCNV” package to extract malignant cells from osteoblasts based on Copy Number Variation (CNV) (**Figure** **2**A).

Malignant cells were extracted using T cells as controls, revealing that most osteoblasts exhibited CNV (Figure 2B). Subsequently, normal and malignant cells in the “Observation” were clustered into 8 classes, indicating that classes 1–3, 5–6, 9, and 13–15 predominantly contained normal cells with the lowest CNV scores. By excluding these classes, 2354 malignant cells were identified (Figure 2C–E).

To further explore the functional differences among cell types in OS samples, we leveraged the “CellChat” R package to investigate pathway activities between different cells. Figure 2F,G illustrate the cell‐cell communication quantity and interaction strength among 10 cell subgroups, highlighting that CAF and malignant cells exhibit higher interaction numbers and intensity (Figure 2H). This suggests a close interaction between OS cells and CAF cells, implicating a potential association between the malignant progression of OS and CAF. Multiple studies have indicated that CAF plays a significant role in causing poor treatment responses in OS.^[^ ^42^, ^43^ ^]^

Single‐cell sequencing analysis indicates a significant signaling interaction intensity between CAF cells and OS malignant cells, suggesting that CAF cells may influence chemotherapy resistance in the tumor microenvironment through various pathways, which has not been extensively explored in previous OS studies.^[^ ^36^ ^]^ Specifically, CAF cells can enhance the survival capacity of malignant cells by secreting oxidative stress molecules and pro‐autophagy signals.^[^ ^44^, ^45^ ^]^ Furthermore, CAF cells directly regulate drug resistance in malignant cells by transferring resistance‐associated non‐coding RNAs via exos.^[^ ^46^ ^]^ The close interaction between CAF cells and malignant cells highlights CAF cells as a potential therapeutic target for overcoming drug resistance and provides new directions for studying resistance mechanisms in OS.

### 2.3 Reversal of Chemoresistance in OS by hsa_circ_0004872

Dox is a frontline chemotherapeutic agent for treating OS, widely utilized for its broad‐spectrum anti‐tumor activity.^[^ ^47^, ^48^ ^]^ However, the emergence of Dox resistance has posed a significant challenge in clinical treatment, markedly limiting its therapeutic efficacy.^[^ ^49^, ^50^ ^]^ Several studies have confirmed the crucial role of circRNAs in regulating drug resistance in cancer.^[^ ^51^, ^52^, ^53^ ^]^

**Figure 3:**
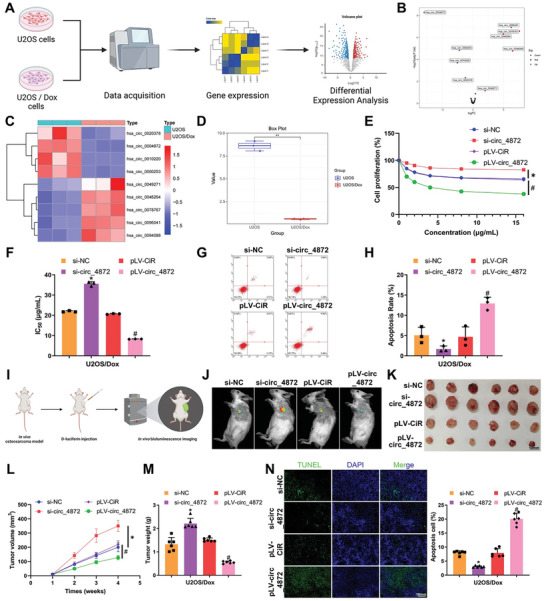
The Impact of hsa_circ_0004872 on Chemoresistance in OS. Note: A) Transcriptomic sequencing analysis workflow of Dox‐resistant cells (Created by Biorender). B) Volcano plot showing differential expression of circRNAs between three U2OS cells and three U2OS/Dox cells in high‐throughput sequencing data. C) Heat map revealing the differential expression of nine circRNAs in the sequencing data. D) Expression profile of hsa_circ_0004872 in high‐throughput sequencing data. E) Viability of U2OS/Dox cells assessed by CCK8 assay. F) IC_50_ values of U2OS/Dox cells. G,H) Flow cytometry analysis of apoptosis in U2OS/Dox cells in each group. I,J) Tumor growth in U2OS/Dox xenograft mice monitored by bioluminescence intensity at different time points, with one representative example shown for each group. K) Morphology of tumor tissues from U2OS/Dox xenograft mice in each group. L) Tumor growth in U2OS/Dox xenograft mice in each group. M) Weight of tumor tissues in U2OS/Dox xenograft mice in each group. N) TUNEL assay showing apoptosis in tumor tissues of U2OS/Dox xenograft mice, with green indicating apoptotic cells and DAPI staining cell nuclei (scale bar: 10 µm). Cell experiments were repeated at least three times with six mice per group. * indicates *p* < 0.05 compared to the si‐NC group, # indicates *p* < 0.05 compared to the PLV‐CIR group.

To identify the key circRNA involved in regulating Dox resistance in OS, we established an in vitro model of chemoresistant OS (**Figure** **3**A). Results demonstrated that prolonged exposure to varying concentrations of Dox induced significant resistance in human OS cell lines. Compared to the untreated parental cells, the IC_50_ values of Dox increased from 2.038 to 21.97 µg mL^−1^ in U2OS resistant cells and from 2.041 to 20.79 µg mL^−1^ in MG‐63 resistant cells (Figure S7A‐D, Supporting Information). These data signify the successful establishment of a chemoresistant OS cell model, with U2OS/Dox displaying better resistance, suitable for subsequent transcriptomic sequencing analysis. Differential expression analysis of the sequencing results revealed a total of nine differentially expressed circRNAs, including five upregulated and four downregulated circRNAs (Figure 3B,C). Among these, hsa_circ_0004872 was significantly downregulated in U2OS/Dox cells, exhibiting the most pronounced difference (Figure 3D). These results suggest that hsa_circ_0004872 may play a critical role in mediating chemoresistance in OS.

In order to determine the impact of circ_0004872 on the anti‐tumor efficacy of Dox, we constructed models of circ_0004872 knockdown and circ_0004872 overexpression in chemoresistant cells, selecting the most effective si‐circ_4872#1 for further experiments (Figure S7E‐H, Supporting Information). Subsequently, treating the OS cells with different concentrations of Dox revealed that after circ_0004872 knockdown, cell viability and IC_50_ values were significantly higher compared to the control group. Furthermore, overexpression of circ_0004872 in OS cells resulted in decreased cell viability and lower IC_50_ values compared to the control group (Figure 3E,F; Figure S7I,J, Supporting Information). Additionally, apoptosis levels were assessed, indicating a significant decrease in apoptosis in the si‐circ_4872 knockdown group, while overexpression of circ_0004872 in the pLV‐circ_4872 group led to a significant increase in apoptosis (Figure 3G,H; Figure S7K,L, Supporting Information).

To further validate the influence of circ_0004872 on Dox's anti‐tumor effects in vivo, we established an animal model by subcutaneous injection of circ_0004872 knockdown cells and initiated treatment with PBS solution or 5 mg kg^−1^ of Dox via intraperitoneal injection starting from the 7th day. The results indicated that overexpression of circ_0004872 sensitized OS cells to Dox treatment, resulting in decreased tumor volume, reduced tumor weight, and significantly increased apoptosis. Conversely, circ_0004872 knockdown enhanced resistance to Dox in OS cells, leading to significantly increased tumor volume, weight, and decreased apoptosis (Figure 3I–N; Figure S8A‐E, Supporting Information). In conclusion, these findings suggest that hsa_circ_0004872 knockdown confers Dox resistance to OS cells, while hsa_circ_0004872 overexpression renders cells more sensitive to Dox.

### 2.4 Reversal of Chemoresistance in OS by the Peptide Encoded by hsa_circ_0004872

Prior studies have suggested that circRNAs containing Internal Ribosome Entry Sites (IRESs) or extensive methylation sites may influence physiological processes by encoding proteins.^[^ ^54^, ^55^ ^]^ Notably, the 109 amino acid peptide encoded by hsa_circ_0004872 has been shown to inhibit cancer progression.^[^ ^56^ ^]^

**Figure 4:**
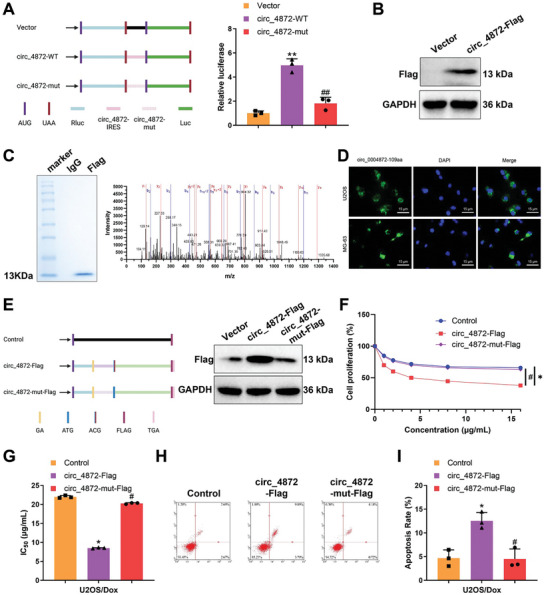
Impact of Small Peptides Encoded by hsa_circ_0004872 on Chemoresistance in OS. Note: A) Cloning of wild‐type or mutant IRES between the Rluc and Luc reporter genes, featuring independent start (AUG) and stop (UGA) codons (left); detection results of relative luciferase activities in each group (right); B) Western blot analysis using Flag antibody to examine the expression of novel proteins encoded by hsa_circ_0004872 overexpression vector (bearing Flag tag); C) Purification of novel proteins from 293T cells transfected with circ_4872‐Flag through co‐immunoprecipitation with Flag antibody, followed by SDS‐PAGE separation, showing gel image of purified proteins stained with Coomassie Brilliant Blue on the left, and peptide sequence results identified by LC‐MS/MS analysis of these novel proteins on the right; D) Immunofluorescence staining of OS cells transfected with Flag‐tagged circ_0004872‐109aa using anti‐Flag antibody to demonstrate the cellular localization of circ_0004872‐109aa (scale bar: 15 µm); E) Schematic representation of a mutant circ_4872‐Flag vector lacking protein‐coding capability (circ_4872‐mut‐Flag) on the left, with Western blot experiment on 293T cells transfected with control, circ_4872‐Flag, and circ_4872‐mut‐Flag vectors on the right to detect the expression of circ_0004872‐109aa protein; F) CCK8 assay to measure the viability of U2OS/Dox cells; G) IC_50_ values of U2OS/Dox cells; H,I) Flow cytometry analysis of apoptosis in various groups of U2OS/Dox cells. Cell experiments were repeated at least three times, with 6 mice in each group. * denotes *p* < 0.05 compared to the Control group, # denotes *p* < 0.05 compared to the circ_4872‐Flag group.

To verify the predicted IRES activity within circ_0004872, a dual‐luciferase assay was conducted, revealing a significant increase in luciferase activity of the wild‐type IRES reporter gene compared to the mutant IRES reporter gene (**Figure** **4**A). To confirm the protein‐coding capability of circ_0004872, a FLAG‐tagged circ_0004872 overexpression vector (circ_4872‐Flag) was constructed just before the last nucleotide of the entire sequence (i.e., the predicted stop codon of circ_0004872) and transfected into 293T cells. Western blot results demonstrated the expression of a novel protein detectable by the FLAG antibody, indicating the protein‐coding ability of circ_0004872 (Figure 4B). Subsequently, the newly synthesized protein was purified by IP using the anti‐FLAG antibody for further verification. LC‐MS/MS results showed that the amino acid sequence of the newly synthesized protein was in agreement with the amino acid sequence of circ_0004872‐109aa^[^ ^56^ ^]^ (Figure 4C). Immunofluorescence experiments confirmed that circ_0004872‐109aa was mainly located in the cytoplasm of OS cells (Figure 4D).

Furthermore, a mutant circ_0004872 overexpression vector lacking the ability to encode the circ_0004872‐109aa protein (circ_4872‐mut‐Flag) was constructed and compared with the linear form circ_4872‐Flag vector. Western blot analysis revealed that circ_4872‐Flag produced the circ_0004872‐109aa protein as detected by the Flag antibody; however, this function was lost in the circ_4872‐mut‐Flag group (Figure 4E). Treatment of OS cells with different concentrations of Dox revealed a significant decrease in cell viability and IC_50_ values in the circ_4872‐Flag group compared to the Control group; conversely, the circ_4872‐mut‐Flag group showed a significant increase in cell viability and IC_50_ values compared to the circ_4872‐Flag group (Figure 4F,G; Figure S9A,B, Supporting Information). Subsequent assessment of apoptosis levels in the cells demonstrated a significant upregulation of apoptosis in the circ_4872‐Flag group compared to the Control group and a significant decrease in apoptosis levels in the circ_4872‐mut‐Flag group compared to the circ_4872‐Flag group (Figure 4H,I; Figure S9C,D, Supporting Information).

To further validate whether circ_0004872‐109aa influences the anti‐tumor effect of Dox in vivo, animal models were developed through subcutaneous injection of cells overexpressing circ_4872‐Flag and circ_4872‐mut‐Flag, followed by Dox treatment. The results revealed increased sensitivity of OS cells in the circ_4872‐Flag group to Dox treatment (Figure S9E,F, Supporting Information), accompanied by reduced tumor volume (Figure S9G‐J, Supporting Information) and tumor weight (Figure S9K,L, Supporting Information), as well as a significant increase in cell apoptosis (Figure S9M,N, Supporting Information), supporting this notion. These findings collectively demonstrate that the peptide encoded by hsa_circ_0004872 can reverse chemoresistance in OS.

### 2.5 Small Peptides Encoded by hsa_circ_0004872 Promote Autophagy‐Dependent Ferroptosis in OS Cells

**Figure 5:**
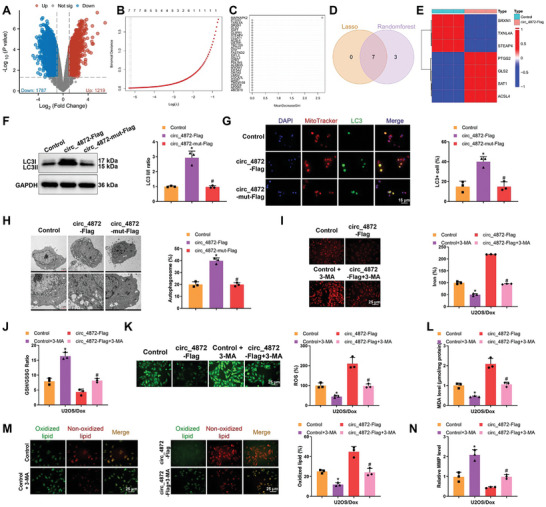
The Influence of Small Peptides Encoded by hsa_circ_0004872 on Autophagy‐Dependent Ferroptosis in U2OS/Dox Cells. Note: A) Differential mRNA volcano plot between U2OS cells transfected with control vector (Control) and U2OS cells transfected with hsa_circ_0004872 overexpression vector (circ_4872‐Flag) based on high‐throughput sequencing data; B) Lasso coefficient selection plot; C) Random forest algorithm results plot; D) Venn diagram showing the intersection of mRNA targets related to small peptides encoded by hsa_circ_0004872 selected by lasso regression and random forest algorithm; E) Heat map showing the expression profile of intersecting genes in the sequencing data; F) Western blot analysis of mitochondrial autophagy‐related protein LC3I/II expression levels in different U2OS cell groups; G) Confocal microscopy observation of mitochondrial and LC3 co‐staining results in M2 macrophages of different groups (scale bar: 15 µm); H) TEM observation of mitochondrial morphology in cells (Scale bar = 2 µm/1 µm); I) Determination of intracellular iron levels in U2OS cells using FerroOrange (Scale bar = 25 µm); J) GSH/GSSG ratio in U2OS cells after different treatments; K) Visualization of ROS production in U2OS cells using DCFH‐DA under confocal laser scanning microscope and statistical analysis by flow cytometry, Scale bar = 25 µm; L) Measurement of MDA levels in each group; M) Representative confocal images of C11‐BODIPY 581/591‐stained U2OS cells displaying oxidized lipids in green and non‐oxidized lipids in red (Scale bar = 25 µm); N) Detection of mitochondrial membrane potential (MMP) in different U2OS cell groups using JC‐1. * indicates *p* < 0.05 compared to the Control group, # indicates *p* < 0.05 compared to the circ_4872‐Flag group, and cell experiments were repeated at least three times.

To investigate the downstream regulatory pathways and mechanisms of chemoresistance in OS influenced by circ_0004872‐109aa, we performed high‐throughput sequencing on U2OS/Dox cells transfected with control vector (Control) and circ_0004872‐109aa overexpression vector (circ_4872‐Flag). A total of 3012 DEGs were identified (**Figure** **5**A). The GO enrichment analysis revealed that these genes were significantly enriched in ion channel activity, gated channel activity, and metal ion transmembrane transporter activity, among other molecular functions. Additionally, the KEGG enrichment analysis indicated significant enrichment in pathways related to cancer, proteoglycans in cancer, and ferroptosis among others (Figure S10, Supporting Information). Through LASSO analysis, seven core genes were identified (Figure 5B). Furthermore, the assessment of the importance of DEGs using the random forest algorithm led to the identification of the top 10 most important genes (Figure 5C). Subsequently, seven key mRNA transcripts were obtained (Figure 5D), with TXNL4A, SRXN1, and STEAP4 showing downregulation, while ferroptosis‐related genes PTGS2, GLS2, SAT1, and ACSL4 were upregulated in the circ_0004872‐109aa overexpression group (Figure 5E). These bioinformatics findings suggest a close association between circ_0004872‐109aa and the ferroptosis pathway, displaying a positive regulatory relationship.

To further validate the bioinformatics results above, we examined ferroptosis‐specific markers in U2OS/Dox cells with higher drug resistance. The results showed that, compared to the Control group, the levels of Fe2+, ROS, MDA, and lipid peroxidation were significantly increased, while the GSH/GSSG ratio was significantly decreased in both the Erastin‐treated group and the circ_4872‐Flag group. In contrast, compared to the circ_4872‐Flag group, the levels of Fe2+, ROS, MDA, and lipid peroxidation were markedly reduced, and the GSH/GSSG ratio was significantly increased in the circ_4872‐mut‐Flag group (Figure S11A‐E, Supporting Information). JC‐1 was used to assess mitochondrial membrane potential changes, and the results showed that, compared to the Control group, the mitochondrial membrane potential was significantly reduced in both the Erastin‐treated group and the circ_4872‐Flag group, while it was significantly increased in the circ_4872‐mut‐Flag group compared to the circ_4872‐Flag group (Figure S11F, Supporting Information).

Increasing evidence suggests that ferroptosis is a form of autophagic cell death process,^[^ ^57^ ^]^ and autophagy‐dependent ferroptosis has been demonstrated to positively regulate the chemosensitivity of tumors.^[^ ^58^ ^]^ Therefore, we examined the expression levels of the autophagy‐related protein LC3II/I in U2OS/Dox OS cells overexpressing or mutated for circ_0004872‐109aa. The results revealed that compared to the Control group, cells in the circ_4872‐Flag group showed significantly elevated levels of LC3II/I expression, while the trend was opposite in the circ_4872‐mut‐Flag group, with a marked decrease (Figure 5F). Co‐localization of mitochondria and LC3 was observed using confocal microscopy to monitor changes in mitochondrial autophagy. As shown in Figure 5G, in U2OS/Dox OS cells, mitochondria and LC3 exhibited co‐localization, which decreased with the mutation of circ_0004872‐109aa but increased after overexpression of circ_0004872‐109aa. Electron microscopy observations indicated a decrease in mitochondrial autophagosome formation after mutating circ_0004872‐109aa, while the trend was reversed in the group overexpressing circ_0004872‐109aa (Figure 5H). Additionally, treatment of U2OS/Dox OS cells with the autophagy inhibitor 3‐MA^[^ ^58^, ^59^ ^]^ resulted in a significant inhibition of cell ferroptosis induced by circ_0004872‐109aa (Figure 5I–N).

Furthermore, we investigated the impact of circ_0004872‐109aa on autophagy‐dependent ferroptosis in OS cells and its effect on chemoresistance. Treatment of OS cells with the autophagy inhibitor 3‐MA led to a significant increase in cell viability and IC_50_ values, along with reduced apoptosis (Figure S11G‐I, Supporting Information). These findings collectively indicate that the small peptide encoded by hsa_circ_0004872 can promote autophagy‐dependent ferroptosis in OS cells, thereby reversing their chemoresistance.

### 2.6 NGR‐109aa‐Exos Enhance Autophagy‐Dependent Ferroptosis in OS Cells

**Figure 6:**
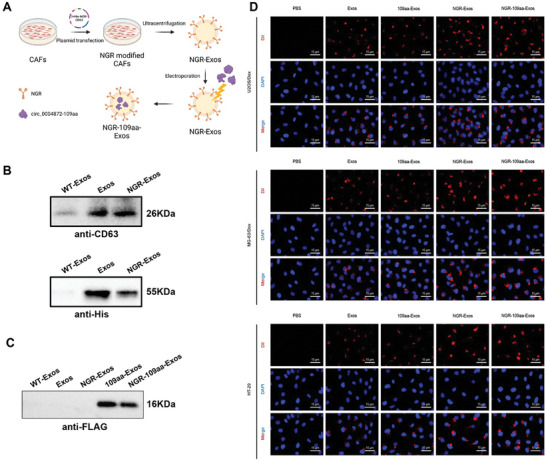
Construction of Tumor Vasculature‐Targeting Peptide NGR‐Modified CAFs Exos Encapsulating circ_0004872‐109aa. Note: A) Schematic diagram of NGR‐109aa‐Exos synthesis (Created by Biorender); B) Transfection efficacy of NGR overexpression vector plasmid detected using anti‐CD63 and anti‐His antibodies; C) Packaging effect of exos on circ_0004872‐109aa under electroporation technique detected using anti‐FLAG antibody; D) The uptake status of EVs by U2OS/Dox, MG‐63/Dox cells and HT‐29 cells observed under laser scanning confocal microscopy, where red fluorescence represents Dil, blue is for DAPI nuclear staining (scale bar: 15 µm). Cell experiments were conducted at least three times.

We have elucidated the mechanism by which circ_0004872‐109aa promotes autophagy‐dependent ferroptosis in OS cells, impacting the chemosensitivity of these cells. In order to address the significant downregulation of circ_0004872‐109aa in tumor tissues, we propose the development of a nano‐material capable of carrying circ_0004872‐109aa targeted delivery to OS cells. Recently, exos derived from CAFs have been discovered to selectively deliver cargo to tumor cells^[^ ^60^ ^]^ with good biocompatibility and low immunogenicity.^[^ ^61^ ^]^ Therefore, following engineering modifications, exos derived from CAFs are gaining attention as carriers for RNA, proteins, and small molecule drugs.^[^ ^12^ ^]^ NGR peptide specifically binds to CD13 on tumor cells and tumor‐related blood vessels, facilitating the precise delivery of cargo to the tumor site, thus reducing the impact on normal tissues.^[^ ^60^, ^62^ ^]^ Results in Figure 2F–H demonstrate close communication between OS cells and CAF cells, leading us to select CAFs‐derived exos for constructing nano‐materials for delivering circ_0004872‐109aa, while utilizing NGR modification on the exos to enhance targeting effectiveness, as depicted in **Figure** **6**A.

Initially, we established stable cell lines of CAFs overexpressing 3xHis‐CD63 and 3xHis‐NGR‐CD63 fusion proteins, from which exos were isolated. Western blot analysis confirmed the expression of classic exo markers (Alix, TSG101, CD81) in the exos derived from CAFs (Figure S12A, Supporting Information). TEM and NTA validated the typical cup‐shaped morphology of exos, ranging in size from 50 to 200 nm (Figure S12B,C, Supporting Information). Through protein imprinting, we identified the fusion proteins 3xHis‐NGR‐CD63 (NGR‐Exos) and 3xHis‐CD63 (Exos) in exos derived from CAF cells. Antibody detection showed that the molecular weight of exos derived from CAFs overexpressing 3xHis‐NGR‐CD63 fusion protein matched that detected by the CD63 antibody, confirming the successful construction (Figure 6B).

Subsequently, utilizing electroporation, we encapsulated circ_0004872‐109aa into exos. FLAG antibody detection revealed the presence of circ_0004872‐109aa in both the 109aa‐Exos group and NGR‐109aa‐Exos group, indicating successful construction of tumor vessel‐targeting peptide NGR‐modified CAFs encapsulating circ_0004872‐109aa (Figure 6C). Additionally, RT‐qPCR was used to assess the loading capacity of the 109aa‐Exos and NGR‐109aa‐Exos groups, revealing that NGR‐modified Exos had a better loading capacity for circ_0004872‐109aa (Figure S12D, Supporting Information). To further examine the uptake of circ_0004872‐109aa or the various Exos groups by colorectal cancer cells HT‐29 and OS cells, each Exos group was labeled with Dil and then co‐incubated with U2OS/Dox and MG‐63/Dox cells for 24 h. Laser scanning microscopy was used to observe Exos uptake, with the results showing no red fluorescence signal in the PBS group OS‐resistant cells, while significant red fluorescence was observed in the other four groups. Notably, the red fluorescence signal was strongest in the NGR‐Exos and NGR‐109aa‐Exos groups, likely due to the targeted effect of NGR enhancing the uptake rate in tumor cells (Figure 6D). These results indicate that all Exos groups were successfully taken up by OS cells.

In conclusion, we have successfully constructed tumor vessel‐targeting peptide NGR‐modified CAFs exos encapsulating circ_0004872‐109aa, which were effectively taken up by OS cells.

### 2.7 NGR‐109aa‐Exos Enhance Autophagy‐Dependent Ferroptosis in OS Cells Reversing Chemoresistance

**Figure 7:**
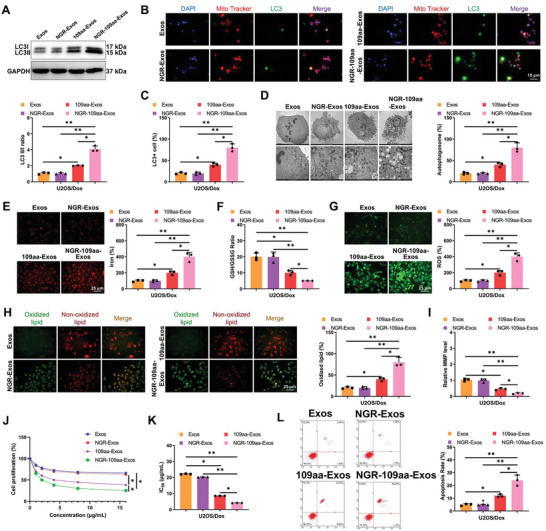
Impact of NGR‐109aa‐Exos on Autophagy‐Dependent Ferroptosis in U2OS/Dox Cells. Note: A) Western blot analysis of mitochondrial autophagy‐related protein LC3I/II expression levels in different U2OS/Dox cell groups; B,C) Confocal laser scanning microscopy observations of mitochondrial and LC3 co‐staining results in M2 macrophages from various groups (scale bar: 15 µm); D) TEM examination of cell mitochondrial morphology; E) Identification of intracellular iron levels in U2OS/Dox cells using FerroOrange (Scale bar = 25 µm); F) GSH/GSSG ratio in U2OS/Dox cells post different treatments; G) Visualization of ROS generation in U2OS/Dox cells using DCFH‐DA under confocal laser scanning microscopy and statistical analysis via flow cytometry, Scale bar = 25 µm; H) Representative confocal images of C11‐BODIPY 581/591 stained U2OS/Dox cells. Red images represent unoxidized lipids, while green depicts oxidized lipids (Scale bar = 25 µm); I) JC‐1 assay to measure mitochondrial membrane potential (MMP) in U2OS/Dox cells; J) CCK8 assay evaluating U2OS/Dox cell viability; K) IC_50_ values for U2OS/Dox cells; L) Flow cytometry analysis of apoptosis in different U2OS/Dox cell groups. *p < 0.05, **p < 0.01, Cell experiments repeated at least three times.

To investigate the impact of NGR‐109aa‐Exos on tumor cell autophagy and ferroptosis, Exos from different groups were co‐incubated with U2OS/Dox cells. The expression levels of the autophagy‐related protein LC3II/I were assessed. The results demonstrated a significant increase in the expression of LC3II/I in cells treated with 109aa‐Exos and NGR‐109aa‐Exos compared to Exos and NGR‐Exos. Additionally, the number of autophagosomes in these cells increased, with the NGR‐109aa‐Exos group showing notably higher levels of LC3II/I and more autophagosomes compared to the 109aa‐Exos group (**Figure** **7**A–D; Figure S13A‐D, Supporting Information).

Evaluation of the ferroptosis‐specific indicators in cells revealed that compared to Exos and NGR‐Exos groups, cells treated with 109aa‐Exos and NGR‐109aa‐Exos exhibited significantly increased levels of Fe^2+^ and ROS content, as well as lipid peroxidation. Moreover, the GSH/GSSG ratio and mitochondrial membrane potential markedly decreased in these groups. Substantial differences in ferroptosis‐related indicators were observed between the NGR‐109aa‐Exos and 109aa‐Exos groups (Figure 7E–I; Figure S13E‐I, Supporting Information).

Subsequently, the therapeutic effect of NGR‐109aa‐Exos on chemotherapy resistance in OS cells was investigated. Treatment with varying concentrations of Dox showed no significant changes in cell viability, IC_50_ values, or apoptosis in the Exos and the NGR‐Exos groups compared to the PBS group. This suggested that the carriers themselves had no apparent toxicity to the cells. Conversely, cells treated with 109aa‐Exos and NGR‐109aa‐Exos exhibited a significant decrease in cell viability and IC_50_ values, along with increased apoptosis compared to the PBS, Exos, and NGR‐Exos groups. Notably, the NGR‐109aa‐Exos group showed a more substantial reduction in cell viability and IC_50_ values than the 109aa‐Exos group, with a significant increase in apoptosis (Figure 7J–L; Figure S13J‐L, Supporting Information). These findings collectively indicate that NGR‐109aa‐Exos can enhance autophagy‐dependent ferroptosis in OS cells, reversing their chemoresistance.

### 2.8 NGR‐109aa‐Exos Reverses Chemoresistance in OS Cells

**Figure 8:**
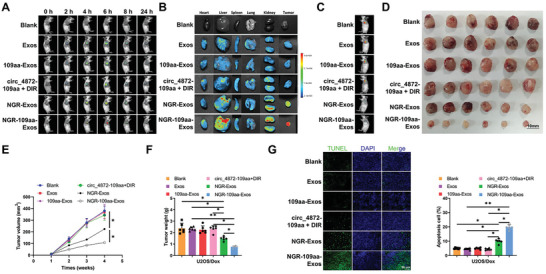
Impact of NGR‐109aa‐Exos on Tumorigenesis of U2OS/Dox Cells In Vivo. Note: A) NIR fluorescence imaging to observe the in vivo fluorescence distribution in tumor‐bearing nude mice in each group; B) NIR fluorescence imaging to observe the fluorescence distribution in major organs and tumor tissues of nude mice in each group; C) Monitoring tumor growth at different time points through bioluminescent intensity measurement, with one representative example shown for each group; D) Morphology of tumor tissues in each group of mice; E) Tumor growth status in each group of mice; F) Tumor tissue weight in each group of mice; G) TUNEL assay to detect cell apoptosis in tumor tissue of each group of mice, with TUNEL staining apoptotic cells in green and DAPI staining cell nuclei in blue (scale bar: 50 µm). *p < 0.05, with 6 mice in each group.

Previous in vitro experiments have demonstrated that NGR‐109aa‐Exos can enhance autophagy‐dependent ferroptosis in OS cells, reversing their chemoresistance. To further investigate the impact of NGR‐109aa‐Exos in vivo on the anti‐tumor effect of Dox, we established an animal model by subcutaneously injecting OS cells and administering Dox treatment. Exos labeled with DIR were intravenously injected into mice, and the biodistribution of exos in each group was evaluated through NIR imaging. Live imaging was conducted within 24 h after intravenous injection of exos, followed by ex vivo imaging of major organs and tumors 24 h later. The results revealed that within 24 h, the fluorescence signals of NGR‐Exos group and NGR‐109aa‐Exos group were predominantly located in the kidneys, liver, and tumor tissues. Due to the non‐targeting nature and smaller particle size of DIR, the Exos group, circ_4872‐109aa + DIR group, and 109aa‐Exos group were quickly metabolized from the kidneys, resulting in a gradual decrease in accumulation at the tumor site. In contrast, the tumor fluorescence signal duration was longer in the NGR‐Exos group and NGR‐109aa‐Exos group, with the strongest intensity observed in these groups, attributable to the targeted accumulation of NGR in the tumor site (**Figure** **8**A,B; Figure S14A,B, Supporting Information). Additionally, combined with previous in vitro experiments, NGR‐109aa‐Exos can promote autophagy‐dependent ferroptosis in OS cells to reverse chemotherapy resistance. Therefore, the NGR‐109aa‐Exos group exhibits stronger tumor‐targeting ability and achieves the most potent tumor inhibitory effect.

Subsequently, we evaluated the role of NGR‐109aa‐Exos in sensitizing chemotherapeutic drug efficacy. The results demonstrated that the Exos group, circ_4872‐109aa + DIR group, and NGR‐Exos group showed no significant therapeutic effect, whereas the NGR‐109aa‐Exos group and 109aa‐Exos group exhibited substantial therapeutic effects, with the NGR‐109aa‐Exos group demonstrating the best therapeutic outcome. The OS cells in the NGR‐109aa‐Exos group were more sensitive to Dox treatment (Figure 8C; Figure S14C, Supporting Information), evident by the reduction in tumor volume (Figure 8D,E; Figure S14D,E, Supporting Information), tumor weight (Figure 8F; Figure S14F, Supporting Information), and a significant increase in cell apoptosis (Figure 8G; Figure S14G, Supporting Information). Although the NGR‐Exos group also induced a certain degree of apoptosis, this may be attributed to the inherent cytotoxicity of high‐concentration exos or non‐targeted effects. Moreover, the apoptosis rate in this group was significantly lower than that in the circ_0004872‐109aa‐loaded exosome group, indicating that the primary apoptotic effect originates from the polypeptide encoded by the circRNA.

Subsequently, major organs were collected after the treatment for H&E staining, and no significant pathological changes were observed (Figure S15A, Supporting Information). We also assessed various biochemical indicators, including liver function markers such as alanine aminotransferase (ALT) and aspartate aminotransferase (AST), as well as kidney function markers like creatinine (CRE) and blood urea nitrogen (BUN), all of which remained within normal ranges (Figure S15B‐E, Supporting Information). These findings indicate that NGR‐109aa‐Exos has good biocompatibility. Collectively, these results demonstrate that NGR‐109aa‐Exos can enhance chemotherapy sensitivity in OS cells by promoting autophagy‐dependent ferroptosis.

## 3 Discussion

Autophagy‐dependent ferroptosis has been confirmed as an effective anti‐tumor mechanism, especially demonstrating significant potential in reversing chemoresistance.^[^ ^35^, ^63^ ^]^ While previous studies have mainly focused on the fundamental mechanisms of ferroptosis, such as cell membrane lipid peroxidation and cellular metabolic dysregulation, this study has further revealed the specific role of the small molecule peptide derived from circ_0004872 in promoting autophagy‐dependent ferroptosis in OS treatment. In comparison to prior research, our study not only unveils a novel target but also experimentally validates the peptide's specific effects in reversing chemoresistance. Furthermore, our findings offer a more in‐depth molecular‐level explanation, elucidating the specific interaction between the autophagic process and ferroptosis, a relationship rarely reported in previous studies.

Previous research has established the critical roles of circRNAs in tumorigenesis, progression, and treatment, particularly in their functions of regulating gene expression and participating in cell signaling.^[^ ^64^, ^65^ ^]^ Yet, the application of circRNA‐encoded small molecule peptides and their delivery via exos in cancer therapy is a novel concept.^[^ ^18^, ^19^, ^20^ ^]^ Our study not only demonstrates the feasibility of this strategy but also enhances the targeting and therapeutic efficacy of treatment molecules by utilizing NGR‐modified CAF exos. Compared to conventional drug delivery systems, using exos as carriers can more effectively shield therapeutic molecules from degradation in the body, presenting superior biocompatibility and targeting, as corroborated by our experimental results.^[^ ^66^, ^67^, ^68^ ^]^

In the field of targeted cancer therapy, NGR peptides have been extensively studied for their high specificity in recognizing tumor‐specific vascular ligands.^[^ ^69^, ^70^ ^]^ In this study, NGR‐modified CAF‐derived exos demonstrated excellent tumor‐targeting ability, which is consistent with previous studies involving NGR. While the use of NGR‐modified exos for targeted tumor delivery has been reported, this study is the first to apply them in promoting autophagy‐dependent ferroptosis in OS cells. Furthermore, we combined NGR peptide delivery with circRNA‐encoded small peptides, exploring a novel dual‐targeting strategy. This approach not only enhanced the targeting specificity of the therapeutic delivery but also improved the stability and bioactivity of therapeutic molecules in the tumor microenvironment via a targeted exosome‐mediated delivery system. Our study reveals the role of the 109aa small peptide encoded by hsa_circ_0004872 in promoting autophagy‐dependent ferroptosis in OS cells, expanding the functional scope of circRNAs and addressing a research gap on the role of circRNA‐encoded small peptides in regulating tumor cell death mechanisms.^[^ ^71^ ^]^ Additionally, we explored the synergistic effect of NGR‐109aa‐Exos with chemotherapy drugs, confirming its ability to significantly enhance chemosensitivity in OS cells. This effect not only effectively overcomes chemotherapy resistance but also offers new insights into optimizing clinical chemotherapy protocols. Although prior studies have applied NGR‐modified exos in various tumor types,^[^ ^72^ ^]^ investigations into OS resistance and chemosensitivity regulation remain limited. Through the innovative application of NGR‐109aa‐Exos, our study fills this gap and provides a new direction for precision OS treatment. Our findings demonstrate the effectiveness of this dual‐targeting strategy in reversing chemotherapy resistance in OS. Notably, while CAF‐derived exos have the natural advantage of accumulating in tumor sites, thus enhancing drug distribution in tumor regions, they may also carry pro‐tumorigenic risks.^[^ ^73^ ^]^ Despite this potential risk, our study demonstrates that modifying these EVs with NGR is an effective therapeutic approach. Additionally, we conducted biocompatibility and safety assessments, and no significant adverse effects were observed.

This study employed in vitro cell models and a subcutaneous tumor transplant model in mice, providing a robust experimental foundation for validating the role of circ_0004872‐encoded peptide in reversing chemo‐resistance. Through in vitro models, we were able to observe in a controlled setting the direct impact of treatment strategies on cellular behavior, such as changes in cell proliferation, migration, and death. Furthermore, in vitro experiments offered a platform for rapid screening of potential therapeutic molecules, allowing evaluation of multiple candidate molecules before advancing to more expensive and complex in vivo experiments. In the in vivo model, the mouse tumor transplant model enabled the assessment of the efficacy of treatment strategies throughout the whole organism, observing the effects of therapeutic molecules on tumor growth, metastasis, and biological distribution. The combination of these models not only ensured comprehensive research results from molecular to systemic levels but also validated the practicality of treatment strategies in actual biological systems. Additionally, the use of models helped us understand the metabolic pathways of therapeutic molecules in vivo, evaluate potential side effects, and provide data support for the design and implementation of future clinical trials.

Our study delved into the mechanism of action of circ_0004872 and its encoded small peptide in regulating autophagy‐dependent ferroptosis in OS cells. Through detailed molecular biology experiments, we revealed how circ_0004872 and its encoded small peptide influence intracellular iron metabolism and the autophagy pathway. The study found that this small peptide could directly or indirectly affect the internalization and release of iron ions, enhance lipid peroxidation, thereby driving iron‐dependent cell death. We also explored the interaction of this small peptide with known ferroptosis‐regulating proteins such as GPX4 and its intracellular distribution. This research not only adds a new dimension to the regulatory network of ferroptosis but also provides a theoretical basis for using circRNA‐encoded peptides as anti‐tumor strategies. Further research could investigate whether this small peptide interacts with other cellular signaling pathways and whether its efficacy is universal across different types of tumors.^[^ ^18^, ^19^, ^20^ ^]^

In this study, the use of high‐throughput sequencing, single‐cell sequencing, and flow cytometry ensured the precision and reliability of the experimental data. High‐throughput sequencing provided extensive genetic information, allowing us to identify chemotherapy resistance‐related circRNAs among thousands of genes. Single‐cell sequencing enabled us to understand the expression and regulation of these circRNAs at the single‐cell level. Specifically, we used scRNA‐seq to analyze the interactions between CAF cells and malignant cells in the OS tumor microenvironment, particularly their potential mechanisms in chemotherapy resistance. This analysis provides new insights into the molecular pathways underlying OS resistance. Unlike previous studies,^[^ ^36^ ^]^ we not only identified the main types of CAF and malignant cells but also analyzed their dynamic interactions in the tumor microenvironment. This in‐depth analysis reveals that CAF cells regulate the drug resistance of malignant cells through specific signaling pathways, potentially impacting therapeutic efficacy. Flow cytometry was used to verify changes in cell phenotype following experimental interventions, such as apoptosis and cell cycle alterations.^[^ ^36^, ^74^, ^75^ ^]^ The combination of these technologies improved data coverage and depth, allowing us to validate hypotheses from multiple perspectives and ensure the reliability and validity of our findings. Additionally, these methods helped us accurately assess the targeting specificity and side effects of the therapeutic strategy, providing a solid scientific foundation for future clinical applications.

This study is the first to confirm that the small peptide encoded by hsa_circ_0004872 can reverse chemotherapy resistance in OS cells by promoting autophagy‐dependent ferroptosis. This discovery not only broadens our understanding of the role of ferroptosis in cancer treatment but also presents a potential new approach for developing anticancer therapies. In this study, we used an immunosuppressive (S‐IS) cell model due to its notable chemotherapy resistance and its clinical prevalence, making it a better mimic of real treatment challenges.^[^ ^76^ ^]^ However, our study has some limitations, such as the choice of model, which may affect the generalizability of the results, as different molecular subtypes of OS may respond differently to chemotherapy. Future studies should verify this strategy in additional tumor models. Furthermore, different results may be obtained when using subcutaneous versus orthotopic tumor models. While this study focuses on a subcutaneous model for proof of concept, further evaluation of the efficacy of the NGR‐modified exosome delivery strategy in more orthotopic OS models is necessary. In future research, to enhance the external validity of the results, we plan to introduce an orthotopic OS model to better simulate tumor growth and drug response at the primary site. Additionally, the use of patient‐derived xenograft (PDX) models will provide more clinically relevant validation of the efficacy of the circ_0004872‐encoded small peptide strategy in various patient contexts. Moreover, our data do not fully elucidate the specific role of the circ_0004872‐derived small peptide in the autophagy‐dependent ferroptosis mechanism. In future studies, we plan to collect tumor samples from each group at the end of experiments and conduct single‐cell sequencing to analyze the peptide's function in different cell populations, clarifying its molecular mechanisms in regulating autophagy and ferroptosis. The single‐cell‐level analysis will help us decode the specific impact of the small peptide within the heterogeneous tumor microenvironment, providing robust support for enhancing OS chemosensitivity. Finally, further investigation is needed on the in vivo distribution, metabolism, and long‐term safety of the therapeutic molecule. Looking forward, we anticipate that these findings will pave the way for clinical applications, ultimately improving chemotherapy efficacy and offering hope to patients with OS.

## 4 Conclusion

**Figure 9:**
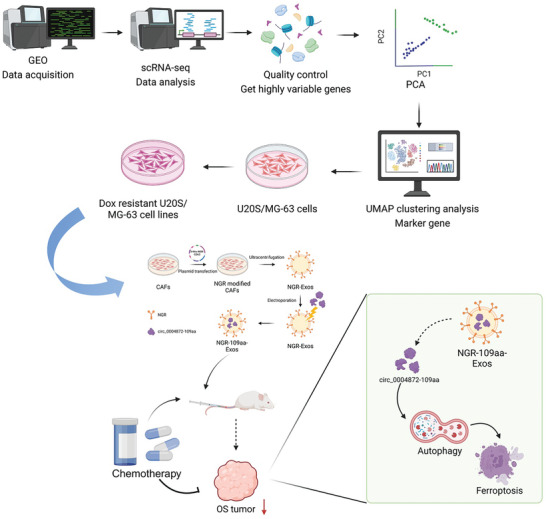
Molecular Mechanism of Reversing Chemotherapy Resistance in OS by Promoting Autophagy‐Dependent Ferroptosis through CAFs‐Derived Exo Delivery of Small Peptide Encoded by circ_0004872 and Modified with Tumor Vascular‐Targeting Peptide NGR (Created by Biorender).

Based on the aforementioned results, we can preliminarily draw the following conclusions: The small peptide encoded by hsa_circ_0004872 can promote autophagy‐dependent ferroptosis in OS cells, thereby reversing their chemoresistance. The tumor vasculature‐targeting peptide NGR modified CAFs‐derived exos encapsulating circ_0004872‐109aa enhances chemosensitivity by promoting autophagy‐dependent ferroptosis in OS cells (**Figure** **9**). This study introduces a novel therapeutic strategy to overcome the chemotherapy resistance of OS that is difficult to tackle with conventional treatments. Our research provides a theoretical basis for elucidating the mechanisms of OS progression and developing new therapeutic targets for OS. However, this study has certain limitations. First, despite demonstrating positive results in in vitro cellular experiments and animal models, the successful translation of these findings into clinical applications still requires further validation. Additionally, the long‐term safety and potential side effects remain unclear; therefore, we will focus on exploring this aspect in our future research endeavors.

## 5 Experimental Section

### Obtaining OS‐Related Single‐Cell Sequencing Data

OS‐related single‐cell RNA sequencing (scRNA‐seq) data was retrieved from the Gene Expression Omnibus (GEO) database using the dataset GSE152048, which comprises tumor tissue samples from 11 OS patients. The data from GSE152048 was analyzed using the “Seurat” package in R software.^[^ ^77^ ^]^ Data quality control was performed based on the criteria of 200 < nFeature_RNA < 5000 and percent.mt < 25, followed by the selection of the top 2000 highly variable gene expressions based on variance.^[^ ^78^ ^]^

### UMAP Clustering Analysis and Cell Annotation

In order to reduce the dimensionality of the scRNA‐Seq dataset, a principal component analysis (PCA) was conducted based on the top 2000 highly variable genes determined by variance. The top 20 principal components were selected for downstream analysis using the Elbowplot function of the Seurat package. The FindClusters function provided by Seurat was utilized to identify main cell subgroups (resolution = 0.5). Subsequently, the UMAP algorithm was applied for non‐linear dimensionality reduction of the scRNA‐seq sequencing data. Cell annotation was carried out by identifying known cell lineage‐specific marker genes and utilizing the CellMarker online tool, with the marker genes for each cell type annotation presented in Table S1 (Supporting Information).^[^ ^79^ ^]^

### Cell Culture and Establishment of Drug‐Resistant Cells

The human OS cell lines U2OS (HTB‐96) and MG‐63 (CRL‐1427) were purchased from ATCC (USA), and the human colon cancer cell line HT‐29 (CL‐0118) was obtained from Procell (China). U2OS, MG‐63, and HT‐29 cells were cultured in McCoy's 5A medium (16600082, Gibco, USA) supplemented with 10% FBS (12484028, Gibco, USA) and 1% penicillin/streptomycin (15140148, Gibco, USA).

The drug‐resistant cell lines U2OS/Dox and MG‐63/Dox were developed using a stepwise intermittent escalation method. Log‐phase U2OS/MG‐63 cells were gradually exposed to increasing concentrations of doxorubicin (Dox; HY‐15142A, MedChemExpress, USA) in the culture medium (30 ng mL^−1^, 100 ng mL^−1^, and 580 ng mL^−1^) for nine cycles at each concentration. Subsequently, the drug concentration was further increased after stable cell growth. Dox induction lasted for 6–8 months until cells were able to grow stably at 580 ng mL^−1^ of Dox (Figure S1, Supporting Information). The IC_50_ values of the resistant cell lines were determined, and the Resistance Index (RI) was calculated as RI = [IC_50_ value of resistant cell line] / [IC_50_ value of parental cell line]. An RI > 10 indicated the successful establishment of the drug‐resistant cell line. Both resistant and parental cell lines were maintained at 37 °C, 5% CO_2_ in a humidified incubator using McCoy's 5A medium with 10% FBS. Prior to experiments, cells were incubated in a drug‐free medium for 3 days.^[^ ^80^ ^]^ In investigating the impact of autophagy‐dependent ferroptosis in drug‐resistant OS cells, 5 mmol L^−1^ of autophagy inhibitor 3‐methyladenine (3‐MA; HY‐19312, MedChemExpress, USA) was added to the culture medium for 24 h before subsequent experiments.^[^ ^58^, ^59^ ^]^

The 293T cell line was obtained from ATCC (CRL‐3216) and cultured in DMEM medium (11965092, Gibco, USA) containing 10% FBS, 10 µg mL^−1^ streptomycin, and 100 U mL^−1^ penicillin. All cells were maintained at 37 °C, 5% CO_2_ in a humidified incubator (Heracell Vios 160i CR CO_2_ Incubator, 51033770, Thermo Scientific, Germany). Passaging was performed when cells reached 80%–90% confluence.^[^ ^81^ ^]^

### circRNA RNA‐Seq

After establishing drug‐resistant OS cell lines, three U2OS and three U2OS/Dox cell samples were collected. Total RNA was extracted using the Total RNA Isolation Reagent Kit (Invitrogen, USA) and quantified by OD value measurements. RNA integrity was verified via agarose gel electrophoresis. Each sample provided 5 µg of total RNA for library preparation. Ribosomal RNA was depleted using the Epicentre Ribo‐zero rRNA Removal Kit (Epicentre, USA), followed by ethanol precipitation to eliminate residual rRNA. Linear RNA was digested with RNase R (Epicentre, USA).

Sequencing libraries were prepared using the NEBNext Ultra II RNA Library Prep Kit for Illumina (NEB, USA) following the manufacturer's protocol. RNA was fragmented under high temperature in the first‐strand synthesis buffer, and cDNA synthesis was performed using random hexamer primers and M‐MuLV Reverse Transcriptase. The second strand was synthesized with DNA Polymerase I and RNase H, substituting dTTP with dUTP. Blunt‐ended DNA fragments were adenylated, ligated to adaptors with hairpin loop structures, and purified using the AMPure XP system (Beckman Coulter, USA). USER enzyme treatment and PCR amplification with Phusion High‐Fidelity DNA Polymerase followed. Libraries were purified with the AMPure XP system, assessed using the Agilent Bioanalyzer 2100 system, and sequenced on the Illumina Hiseq 4000 platform, generating 150 bp paired‐end reads.

Raw Fastq data were processed using a Perl script to remove adapter sequences, poly‐N regions, and low‐quality reads, producing clean reads. Q20, Q30, and GC content metrics were calculated to ensure data quality. The reference genome and annotation files were downloaded, and a genome index was constructed using Bowtie2 v2.2.8. Clean reads were aligned to the reference genome, and circRNAs were identified using find_circ and CIRI2 tools. Expression levels were normalized to TPM using the formula: level = (readCount × 1,000,000) / libsize, where libsize was the total circRNA read count.^[^ ^82^ ^]^

### Enhancing Plasmid Construction and Transfection Efficiency

Transfection of si‐NC and si‐circ_4872 was performed using the lipofection method, with the si‐RNA sequences listed in Table S2 (Supporting Information). For lipofection‐mediated transfection, 5×10^5^ cells were seeded in 6‐well plates. When cell confluence reached 70–90%, transfection was carried out according to the instructions provided by Invitrogen Lipofectamine 3000 (L3000150), mixed with pre‐prepared siRNA containing the luciferase gene to form siRNA‐lipid complexes. After incubating at room temperature for 20–30 min, the siRNA‐lipid complexes were added to the respective wells. Transfected cells were analyzed and further analyzed 2–4 days post‐transfection.^[^ ^83^, ^84^, ^85^ ^]^

To construct the hsa_circ_0004872 overexpression vector pLV‐circ_4872, the hsa_circ_0004872 sequence was inserted into the pLV‐CiR expression vector (KL‐ZL0012‐01, Ke Lei Biological Technology Co., Ltd., China). This vector was constructed and packaged by Genechem (Shanghai, China). To establish a stable overexpression cell line for hsa_circ_0004872, either the control vector pLV‐CiR or the pLV‐circ_4872 vector was transfected into cells using Lipofectamine 3000. The cells were then selected with 10 µg mL^−1^ puromycin for 2–3 weeks until hsa_circ_0004872 stable overexpression was achieved.^[^ ^71^ ^]^ Additionally, for subsequent bioluminescence imaging experiments, the overexpression vector carried the GFP/mCherry genes.

The cell transfection groups were as follows: si‐NC group (OS cells without circ_0004872 knockdown), si‐circ_4872 group (OS cells with circ_0004872 knockdown), pLV‐CiR group (OS cells without circ_0004872 overexpression), and pLV‐circ_4872 group (OS cells with circ_0004872 overexpression).

### Cell Viability Assayed by CCK‐8 Method

Cell viability of OS cells regarding chemoresistance was analyzed following the manufacturer's protocol of the CCK‐8 assay kit (C0041, Beyotime, Shanghai). In brief, cells were seeded into a 96‐well plate at a density of 4 × 10^3^ cells per well. After treatment with varying concentrations (10 to 500 nmol L^−1^) of Dox for 24 h, 10 µL of CCK‐8 solution was added to each well, followed by a 2‐h incubation period. Subsequently, the absorbance at 450 nm was measured using a microplate reader to calculate cell viability, which was determined by the formula: Cell viability = (ΔA_sample – ΔA_blank) / (ΔA_control – ΔA_blank), where ΔA_sample represents the absorbance difference of the sample, ΔA_blank was the absorbance difference of the blank, and ΔA_control was the absorbance difference of the control. The data was curve‐fitted to unveil the relationship between drug concentration and effect, and the IC_50_ value was calculated.^[^ ^86^, ^87^ ^]^

### Flow Cytometry Analysis

The steps for detecting apoptosis in tumor cells using the dual staining reagent kit of membrane protein V‐FITC and propidium iodide (PI) (V13242, Invitrogen, USA) were as follows: the processed tumor cells were collected and washed three times with PBS. Subsequently, these cells were individually stained with 5 µL of membrane protein V and PI for 15 min. Finally, flow cytometry was performed using a flow cytometer (BD Bioscience, BD LSRFortessa, USA) for result analysis. Data analysis was carried out using CellQuest software (BD Biosciences, USA), where the lower‐left quadrant indicates normal cells, the upper‐left quadrant signifies necrotic cells, the upper‐right quadrant indicates late‐stage apoptosis and the lower‐right quadrant represents early‐stage apoptotic cells.^[^ ^88^, ^89^ ^]^

### Animal Experiments In Vivo

Male NOD/SCID mice (6–8 weeks old, 20–30 g) were purchased from Hunan Sleek Jingda Experimental Animal Co., Ltd., China, and housed individually in SPF‐level facilities. Environmental conditions were maintained at 60–65% humidity, a temperature of 22–25 °C, and a 12‐h light‐dark cycle. The mice were provided ad libitum access to food and water. After a one‐week acclimatization period, their health status was assessed before the start of the experiments. All procedures were performed in compliance with the Guidelines for the Care and Use of Laboratory Animals^[^ ^90^, ^91^ ^]^ and were approved by the institutional ethics committee (Approval No. 2024037).

Subsequently, 5 × 10⁶ U2OS/Dox or MG‐63/Dox cells were subcutaneously injected into the left axillary region of the mice. Tumor growth was monitored and photographed (Figure S2, Supporting Information). Tumor volume was measured every other day, and once tumors reached ≈3 mm in diameter, Dox was administered intraperitoneally at 5 mg kg^−1^ twice weekly. Two weeks after drug administration, the bioluminescence signal of pretreated MG‐63/Dox xenografts was analyzed using the CRi Maestro in vivo imaging system (Cambridge Research & Instrumentation, Massachusetts, USA). After anesthesia with 2% isoflurane, mice received intraperitoneal injections of D‐luciferin (150 mg kg^−1^; 122799, PerkinElmer, USA), and images were captured at 15‐minute intervals with a 10‐minute exposure time.^[^ ^92^ ^]^ Tumor growth curves were generated by measuring tumor volume every 7 days post‐injection using the formula: (a × b^2^) / 2, where ‘a’ was the longest diameter and ‘b’ the shortest. Mice were euthanized by CO₂ asphyxiation 28 days post‐injection, after which tumors were excised, weighed, and analyzed.^[^ ^86^, ^93^ ^]^

The mice were randomly divided into 13 groups (n = 6 per group): (1) si‐NC group; (2) si‐circ_4872 group; (3) PLV‐CiR group; (4) PLV‐circ_4872 group; (5) Control group; (6) circ_4872‐Flag group; (7) circ_4872‐mut‐Flag group; (8) Blank group; (9) Exos group; (10) NGR‐Exos group; (11) circ_4872‐109aa + DIR group; (12) 109aa‐Exos group; (13) NGR‐109aa‐Exos group. All groups received Dox treatment. When tumor reached 50 mm^3^ in size, 100 µL of DIR (40757ES25, YEASEN, China) labeled Exos (100 µg in 50 µg/100 µL PBS) was intravenously injected into the mice three times a week.^[^ ^94^, ^95^ ^]^ Whole‐body fluorescence imaging was conducted using a near‐infrared (NIR) two‐zone small animal in vivo imaging system (Photon) at 2, 4, 6, 8, and 24 h post‐injection. At 24 h post‐injection, major organs (liver, spleen, kidneys, heart, lungs, and tumors) were dissected, and fluorescence intensity was measured.

Intratumoral Imaging: Tumor tissues were subjected to ex vivo fluorescence imaging. Tissues were fixed in 4% paraformaldehyde for 24 h, incubated in a 15% sucrose PBS solution for an additional 24 h until sedimentation, then transferred to a 30% sucrose solution for another 24 h. Subsequently, tumor tissues were sectioned to into 20 µm slices, stained with 1 mg mL^−1^ DAPI for 10 min at room temperature, washed twice with PBS (pH 7.4), and immediately examined under a laser scanning confocal microscope (CLSM; LSM 700, Carl Zeiss Microscopy, Germany).^[^ ^96^, ^97^ ^]^

### Histopathological Staining

Hematoxylin and Eosin (H&E) staining: Tissue samples were fixed, sliced, and deparaffinized in xylene. Sections were rehydrated in graded ethanol (100%, 95%, and 70%), washed with water, and mounted. Hematoxylin staining (H8070, Solarbio, Beijing, China) was applied for 5–10 min at room temperature, followed by differentiation in 1% hydrochloric acid ethanol solution for 10 seconds. Sections were washed, dehydrated in 95% ethanol, and immersed in eosin staining solution (G1100, Solarbio, Beijing) for 5–10 min. Routine dehydration, clearing, and mounting were subsequently performed.^[^ ^98^ ^]^

### TUNEL Staining

Tissues were fixed in 4% paraformaldehyde for 15 min, permeabilized with 0.25% Triton X‐100 for 20 min, and blocked with 5% bovine serum albumin (BSA, 36101ES25, Yeasen Biotechnology, Shanghai, China). Samples were stained using a TUNEL kit (C1086, Beyotime Biotechnology, Shanghai, China). DAPI staining solution (C1002, Beyotime, Shanghai) was applied for nuclear restaining. Apoptotic cells (green fluorescence) and total nuclei (blue fluorescence) were imaged using a confocal microscope (LSM 880, Carl Zeiss AG, Germany). Apoptosis rates were calculated as the percentage of apoptotic cells to total cells.^[^ ^99^ ^]^

### Fluorescence Reporter Gene Assay

Plasmids containing renilla luciferase (Rluc) upstream and firefly luciferase (Luc) downstream were constructed by Genechem (Shanghai, China). Potential IRES sequences of hsa_circ_0004872 and its mutants were inserted between Rluc and Luc. Transfections were performed using Lipofectamine 3000, and luciferase activity was measured after 48 h using the Duo‐Luciferase HS Assay Kit (LF004, GeneCopoeia, USA). Results were expressed as Luc/Rluc activity.^[^ ^100^, ^101^ ^]^

### Tagged RNA Affinity Purification (TRAP) Assay

MS2 stem‐loop structures were incorporated into vectors containing hsa_circ_0004872, its mutants, and GST‐MS2 constructs. The vectors were co‐transfected into OS cells to obtain GST‐MS2‐circRNA complexes, which were precipitated using glutathione (GSH) magnetic beads. CircRNA‐bound proteins were identified via mass spectrometry and validated using Western blot analysis.^[^ ^102^ ^]^

### LC‐MS/MS

Protein samples were analyzed using liquid chromatography‐tandem mass spectrometry (LC‐MS/MS) to generate raw data files. The raw files were processed using the Byonic software, with protein identification conducted against the UniProt‐Homo sapiens database.^[^ ^102^ ^]^

### Subcellular Localization Analysis of Small Peptides Encoded by circ_0004872

The subcellular localization of small peptides encoded by circ_0004872 in OS cells was determined using a Fluorescence in situ Hybridization (FISH) assay kit (C10910, Ruibo Biologicals, China). The peptides were labeled in green, and the nuclei were counterstained with DAPI (C0065, Solarbio, Beijing, China). Images were captured from five randomly selected fields of view using a fluorescence microscope (Olympus, Japan).^[^ ^103^ ^]^

### High‐Throughput Sequencing and Analysis of mRNA in OS Cells

Three U2OS/Dox cell samples transfected with the control vector (Control) and three transfected with the hsa_circ_0004872 overexpression vector (circ_4872‐Flag) were randomly selected for analysis. Total RNA was extracted using the Total RNA Isolation Reagent Kit (12183555, Invitrogen, USA) and quantified using a UV‐visible spectrophotometer (BioSpectrometer basic, Eppendorf, USA). RNA integrity was confirmed via agarose gel electrophoresis. High‐quality RNA was reverse transcribed into cDNA, libraries were prepared, and sequencing was performed on the Illumina NextSeq 500 platform. The raw image data were converted to raw reads using base calling. Adapter sequences and low‐quality reads were removed using cutadapt, resulting in clean reads that were aligned to the human reference genome with Hisat2 software. Gene expression was quantified using R, producing a gene expression matrix.^[^ ^104^ ^]^

Differentially expressed genes (DEGs) were identified using the ‘limma’ R package with |log2FC| > 1 and *P* < 0.05 as selection criteria. Volcano plots and heatmaps were created using the ggplot2 and pheatmap R packages, respectively, while Venn diagrams were generated via the Xiantaozi Academic Database (https://www.xiantaozi.com/). DEGs were further analyzed through Gene Ontology (GO) and Kyoto Encyclopedia of Genes and Genomes (KEGG) pathway enrichment analyses using the SangerBox database (http://sangerbox.com/home.html).^[^ ^105^, ^106^, ^107^ ^]^

### Detecting Intracellular Fe^2+^ Levels

Intracellular Fe^2+^ levels were assessed using the FerroOrange probe (F374, Dojindo, Japan). Pre‐treated OS cells were seeded into confocal culture dishes, washed with Hank's balanced salt solution (HBSS; 13150016, Gibco), and then incubated with 1 µM FerroOrange for 30 min. Fluorescence signals were visualized using a confocal laser scanning microscope (LSM780, Zeiss).^[^ ^108^ ^]^ Additionally, intracellular and tissue iron levels were quantified using an iron assay kit (ab83366, Abcam) according to the manufacturer's protocol (ab83366, Abcam).^[^ ^109^ ^]^

### Transmission Electron Microscope

OS cells were prepared for transmission electron microscopy (TEM). Samples were fixed in 2.5% glutaraldehyde at 4 °C overnight, followed by 1–2 h of fixation in 1% osmium tetroxide. Dehydration was performed using graded ethanol (50%–95%), followed by pure acetone treatment. Samples were infiltrated with embedding resin overnight, polymerized at 70 °C, and sectioned into 70–90 nm slices using a Reichert ultramicrotome. Sections were stained with lead citrate and uranyl acetate and examined under a TEM.^[^ ^109^ ^]^

### Mitochondrial Membrane Potential Measurement

Mitochondrial membrane potential was assessed using the JC‐1 assay kit (40706ES60, YiSheng Biotechnology, Shanghai, China). Cells were incubated with JC‐1 staining solution at 37 °C for 20 min. As a positive control, CCCP (50 µM) was added for 20 min. Cells were washed with JC‐1 buffer and observed under a fluorescence microscope. Monomers were detected at an excitation wavelength of 490 nm and an emission wavelength of 530 nm.^[^ ^110^ ^]^

### Biochemical Analysis

The levels of GSH and oxidized glutathione (GSSG) in cells and tissues were measured using the GSH and GSSG assay kits (S0053, Beyotime Biotechnology, Shanghai). Absorbance was recorded at 450 nm using a microplate reader (Infinite200, Tecan, Beijing), and quantification was based on a standard curve.

Cytoplasmic ROS production was evaluated using the DCFH‐DA fluorescent probe from the ROS assay kit (S0033S, Beyotime Biotechnology, Jiangsu). Cells were incubated with 10 µM DCFH‐DA for 20 min in the dark, digested, centrifuged, and resuspended for flow cytometry analysis (FC500ML, Beckman, USA). For visualization, cells were stained with DCFH‐DA and imaged under a confocal laser scanning microscope (LSM780, Zeiss) at excitation/emission wavelengths of 488/525 nm. Although DCFH‐DA provides useful ROS detection, its precision for H₂O₂ detection was limited. Tissue ROS levels were analyzed using the DHE probe (BH‐02×9621, BoHu Biotechnology, Shanghai) with fluorescence intensity measured in a microplate reader (excitation 488–535 nm, emission 610 nm).

Lipid peroxidation was assessed using the C11‐BODIPY581/591 assay (D3861, Invitrogen, USA), and MDA levels were measured using an MDA assay kit (BC0025, Solarbio, Beijing). Cells were stained with 5 µM C11‐BODIPY581/591 for 15 min in the dark, and fluorescence shifts from red to green were imaged under a fluorescence microscope, reflecting oxidation levels. MDA quantification was performed by measuring absorbance at 532 nm, 600 nm, and 450 nm with a microplate reader. Cells treated with 5 µM Erastin (MCE, HY‐15763) for 24 h were used as controls.^[^ ^108^, ^109^, ^111^ ^]^ For the above biochemical assays, cells treated with 5 µM Erastin (MCE, HY‐15763) for 24 h were used as controls.

### Observation of Mitophagy Using Confocal Immunofluorescence Microscopy

OS cells were stained with 400 nM MitoTracker Deep Red (M22426, Invitrogen, USA) at 37 °C for 30 min, then fixed in 4% paraformaldehyde and permeabilized with 0.2% Triton X‐100. After blocking with 1% BSA in PBS, cells were incubated overnight at 4 °C with anti‐LC3 antibody (PA1‐46286, Invitrogen, USA) and subsequently with Alexa Fluor 488‐conjugated secondary antibody (A‐11008, Invitrogen, USA). Nuclei were counterstained with 300 nM DAPI (D1306, Invitrogen, USA). Imaging was performed using a confocal laser scanning microscope (LSM 700, Carl Zeiss Microscopy, Germany).^[^ ^112^ ^]^

### Isolation and Identification of CAF Cells

Tumor tissues from OS mouse models were enzymatically dissociated using the Tumor Dissociation Kit (130‐096‐730, Miltenyi, Germany) and a gentle MACS Dissociator. The suspension was filtered, and tumor‐associated fibroblasts (CAFs) were isolated using the Tumor‐Associated Fibroblast Isolation Kit (130‐116‐474, Miltenyi). The morphology of isolated CAFs was observed via TEM (Figure S3A, Supporting Information). Cells were stained with anti‐α‐SMA and anti‐Vimentin antibodies followed by fluorescently labeled secondary antibodies. Nuclear staining was performed with DAPI, and microscopy revealed a CAF purity of >95% (Figure S3B, Supporting Information).^[^ ^113^ ^]^

### Isolation and Preparation of Exos

CAF‐derived exos were isolated following transfection with plasmids encoding 3xHis‐NGR‐CD63 or 3xHis‐CD63 (Genechem, Shanghai, China). Transfected CAF cells were cultured in serum‐free IMDM medium (12440053, Gibco, USA) for 48 h at 37 °C with 5% CO₂. The culture supernatant was collected, centrifuged at 300 g for 10 min to remove debris, and processed through sequential centrifugation at 2000 g for 20 min, 16500 g for 30 min, and ultracentrifugation at 120,000 g for 70 min. The pellets were washed and resuspended in PBS. The stability of the 3xHis‐NGR‐CD63 fusion protein in exos was confirmed using His and CD63 antibodies.^[^ ^114^ ^]^

The circ_0004872‐109aa was encapsulated into CAFs‐Exos using electroporation. The purified CAFs‐Exos obtained from earlier centrifugation were resuspended in electroporation buffer containing 1.15 mM potassium phosphate (pH 7.2), 25 mM potassium chloride, and 21% OptiPrep working solution (D1556, Sigma‐Aldrich, USA). The suspension was filtered through a 0.22 µm filter to remove debris. Subsequently, the purified circ_0004872‐109aa protein was added to the exos at a weight ratio of 1:5 to form exo‐circ_0004872‐109aa complexes via electroporation using a Gene Pulser Xcell (Bio‐Rad). Post‐electroporation, the exos were centrifuged at 100,000 g for 2 h at 4 °C, followed by resuspension in cold PBS solution.^[^ ^115^ ^]^

The exos were categorized as follows (Figure S4, Supporting Information): (1) Exos group (CAFs‐derived exos stably expressing 3xHis‐CD63); (2) NGR‐Exos group (CAFs‐derived exos stably expressing 3xHis‐NGR‐CD63); (3) 109aa‐Exos group (CAFs‐derived exos encapsulating circ_0004872‐109aa and stably expressing 3xHis‐CD63); (4) NGR‐109aa‐Exos group (CAFs‐derived exos encapsulating circ_0004872‐109aa and stably expressing 3xHis‐NGR‐CD63).

### Identification of EVs

Isolated EVs were characterized by Western blotting for markers Alix, TSG101, and CD81, with calnexin as a negative control. EV size and concentration were analyzed using nanoparticle tracking analysis (NTA) with the NanoSight LM10 (Malvern). Morphological analysis was conducted by TEM after fixing the EV pellet, followed by dehydration, embedding, and staining with lead and uranyl acetate. TEM imaging confirmed the size and morphology of the EVs.^[^ ^116^, ^117^ ^]^

### Observation of the Uptake of Exos by OS Cells Through Immunofluorescence Staining

Exos derived from CAFs were seeded in a 24‐well plate. Dil dye (C1036, Beyotime Biotechnology Co., Ltd., Shanghai) was added to 40 µg of exos to achieve a final concentration of 25 µM. The mixture was then incubated at room temperature for 30 min, followed by centrifugation at high speed to remove unbound dye. Subsequently, the cells were washed thrice with PBS and fixed with 4% paraformaldehyde (AR1068, BOSTER Biological Technology Co., Ltd., Wuhan) for 30 min. The cell nuclei were stained with DAPI (4’,6‐diamidino‐2‐phenylindole, C1005, Beyotime Biotechnology Co., Ltd., Shanghai) for 30 min, and images of the cells were captured at ×400 magnification using a BX53 fluorescence microscope equipped with a camera (Olympus). Image analysis was performed using ImageJ Pro Plus 6.0 software.^[^ ^118^, ^119^ ^]^

### Relative Expression Levels of Target Genes Detected by RT‐qPCR

Total RNA was extracted from exos, tissues or cells using Trizol reagent (15596026, Invitrogen, USA) and the concentration and purity of the total RNA were assessed at 260/280 nm using NanoDrop LITE (ND‐LITE‐PR, Thermo Scientific, Germany). The extracted total RNA was reverse transcribed into cDNA using the PrimeScript RT reagent Kit with gDNA Eraser (RR047Q, TaKaRa, Japan). Subsequently, the expression of each gene was analyzed by RT‐qPCR using SYBR Green PCR Master Mix reagents (4364344, Applied Biosystems, USA) and the ABI PRISM 7500 Sequence Detection System (Applied Biosystems).

The primers for each gene were synthesized by TaKaRa (Table S3, Supporting Information), with GAPDH serving as the reference gene. The relative expression levels of each gene were determined using the 2^−ΔΔCt^ method, where ΔΔCt = (average Ct value of the target gene in the experimental group – average Ct value of the reference gene in the experimental group) – (average Ct value of the target gene in the control group – average Ct value of the reference gene in the control group). All RT‐qPCR analyses were performed in triplicate.^[^ ^120^, ^121^, ^122^ ^]^

### Western Blot

The process began with the collection of tissues or cells, followed by lysis using an enhanced RIPA lysis buffer containing protease inhibitors (P0013B, Beyotime Biotechnology Co., Ltd, Shanghai, China), and subsequent determination of protein concentration with the BCA protein quantification kit (P0012, Beyotime Biotechnology Co., Ltd, Shanghai, China). The proteins were separated by 10% SDS‐PAGE, and transferred onto a PVDF membrane (FFP39, Beyotime Biotechnology Co., Ltd), which was then blocked with 5% BSA (ST023, Beyotime Biotechnology Co., Ltd) at room temperature for 2 h to prevent non‐specific binding. Following incubation with diluted primary antibodies for 1 h at room temperature, all primary antibodies were rabbit anti‐human (for details, refer to Table S4, Supporting Information). The membrane was washed and incubated with an HRP‐conjugated goat anti‐rabbit secondary antibody (ab6721, 1:2000, abcam, UK) for 1 h at room temperature, followed by exposure to Pierce ECL Western blotting substrate (32209, Thermo Scientific, Germany) after mixing equal amounts of solution A and B in the darkroom and applying it onto the membrane. Exposed images were captured using the Bio‐Rad imaging system (BIO‐RAD, USA) and bands in the Western blot images were quantified for grayscale using Image J analysis software, with GAPDH as the internal control. Each experiment was repeated three times.^[^ ^121^ ^]^

### Statistical Software and Data Analysis Methods

Statistical analyses were performed using R software (version 4.2.1) with RStudio (version 2022.12.0‐353) and GraphPad Prism (version 8.0). Quantitative data were expressed as mean ± standard deviation (SD). Sample sizes were determined based on preliminary studies to ensure adequate statistical power. Normality and homogeneity of variance were assessed using the Shapiro‐Wilk test and Levene's test, respectively. Comparisons between two groups were conducted using independent two‐sample t‐tests, while comparisons among multiple groups were analyzed using one‐way ANOVA followed by Bonferroni post hoc tests for pairwise comparisons. For repeated measures over time, two‐way repeated measures ANOVA was applied. Non‐parametric tests, such as the Mann‐Whitney U test or the Kruskal‐Wallis test, were used for data that did not meet parametric assumptions. Statistical significance was defined as a *P*‐value less than 0.05. All figures and statistical plots were generated using GraphPad Prism and R packages, including ggplot2.

### Ethics Approval and Consent to Participate

All animal experiments were approved by the Animal Ethics Committee of Zibo Central Hospital Affiliated to Binzhou Medical University. All animal procedures were carried out in accordance with the guidelines for the care and use of laboratory animals.

## Conflict of Interest

The authors declare no conflict of interest.

## Author Contributions

J.D., X.M., and M.Y. contributed equally to this work and also co‐first authors. J.D., X.M., and M.Y. contributed equally to the conceptualization and experimental design of the study and conducted key experiments, including the single‐cell transcriptome analysis and high‐throughput sequencing. G.C. performed bioinformatics analyses and provided insights into circRNA‐associated chemoresistance mechanisms. J.L. and Z.Z. were responsible for in vitro experiments, including the generation and characterization of NGR‐modified CAF‐derived exosomes. X.W. supported in vivo studies, including therapeutic efficacy assessments in osteosarcoma models. W.H. and M.T. participated in data interpretation and statistical analysis. T.L., S.R., and P.Z. supervised the project, provided critical revisions, and secured funding. All authors contributed to manuscript drafting and approved the final version for submission.

## Supporting information



Supporting Information

## Data Availability

The data that support the findings of this study are available from the corresponding author upon reasonable request.
