# Supplementary material for: NGR‐Modified CAF‐Derived exos Targeting Tumor Vasculature to Induce Ferroptosis and Overcome Chemoresistance in Osteosarcoma
Source: Adv Sci (Weinh). 2025 Jan 31;12(12):2410918. doi: 10.1002/advs.202410918 (PMC11948032; doi:10.1002/advs.202410918)
Supplement: Supplementary file 1 — Supporting Information [file ADVS-12-2410918-s001.docx]

**
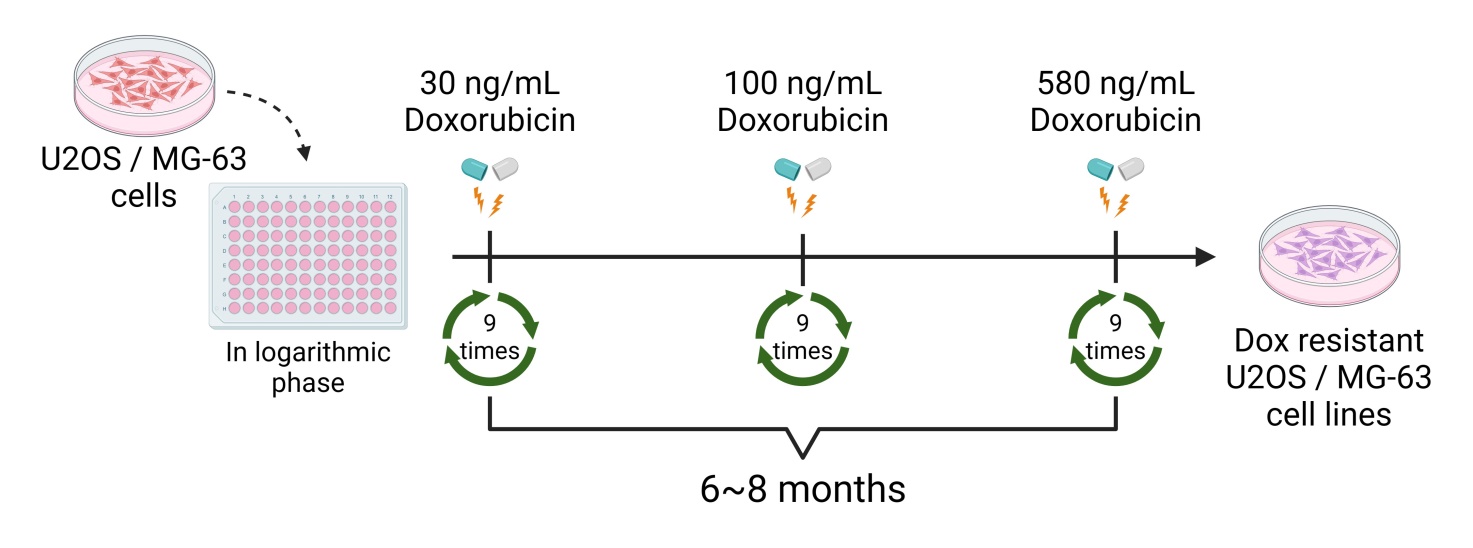
**

**Figure S1. Schematic Diagram of Drug-Resistant OS Cell Line Establishment.**

Note: U2OS/MG-63 cells were used to establish a drug-resistant OS cell line by gradually increasing the concentration of Dox in the culture medium. The cells were cycled through 9 rounds of cultivation at each concentration to develop the drug-resistant cell line (Created by Biorender).

**
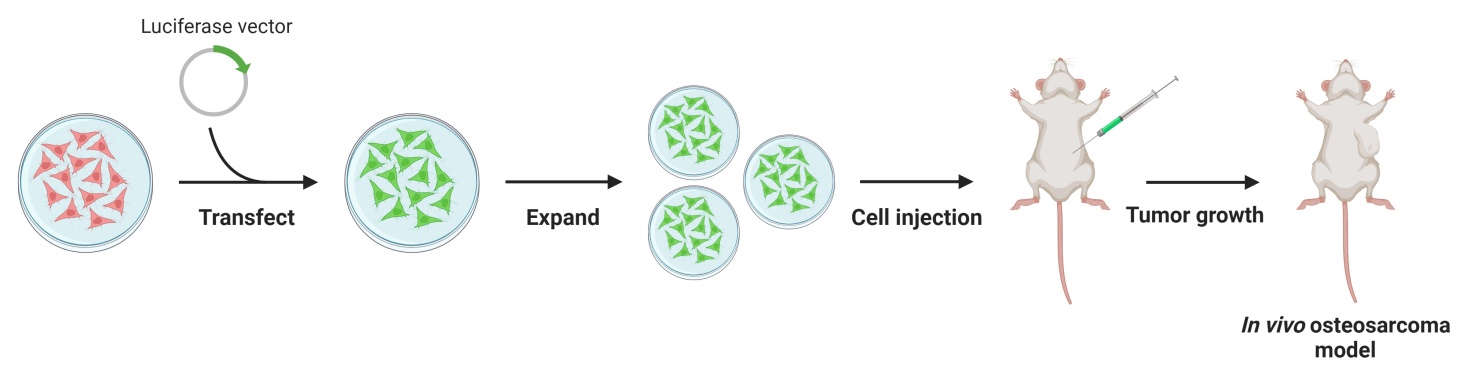
Figure S2. Schematic Diagram of the OS *In Vivo* Animal Model Construction**Note: OS cells were transfected with lentivirus carrying the luciferase gene. These cells were then subcutaneously injected into mice to construct the *in vivo* OS model (Created by Biorender).

**
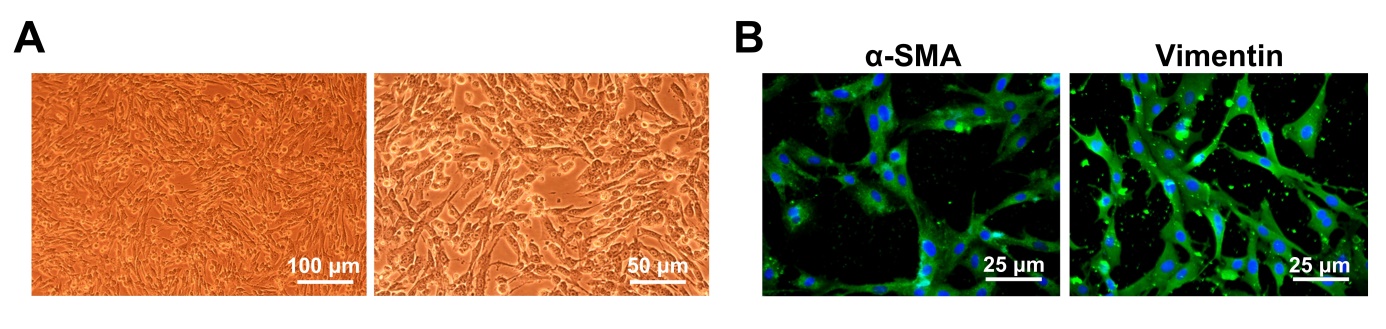
**

**Figure S3. Identification Results of CAFs.**

Note: (A) Representative morphological appearance of CAFs isolated from OS xenograft mice, scale bar: 100/50 μm; (B) Representative immunofluorescence (IF) images of α-SMA and Vimentin in CAFs, scale bar: 25 μm.

**
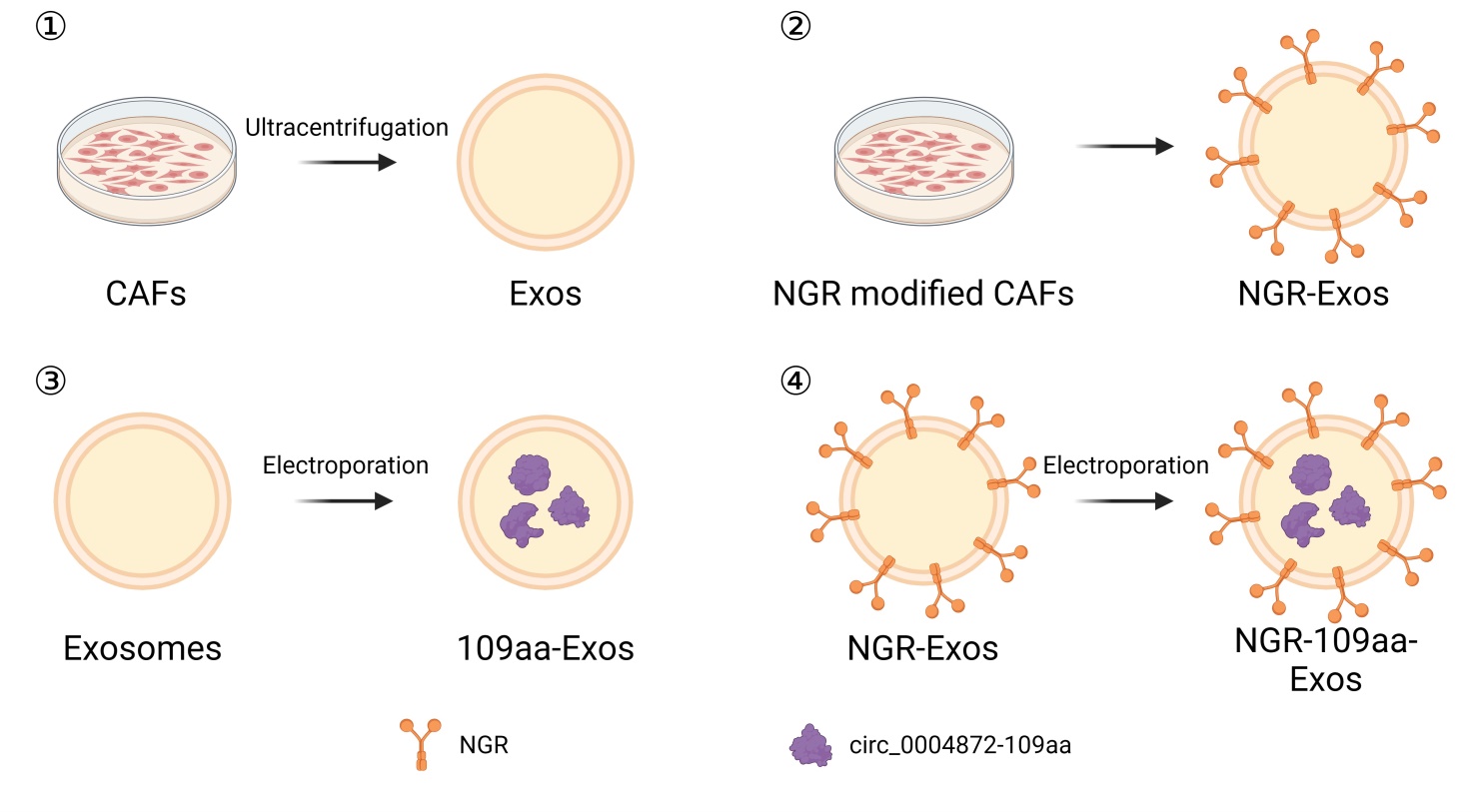
**

**Figure S4. Schematic Representation of Exo Subgrouping.**

Note: ① Illustration of the isolation of exos from CAFs; ② Illustration of the isolation of NGR-exos from CAFs stably expressing 3xHis-NGR-CD63; ③ Schematic representation of the 109aa-exo subgroup obtained through electroporation of exos; ④ Schematic representation of NGR-109aa-exos obtained through electroporation of NGR-exos.

**
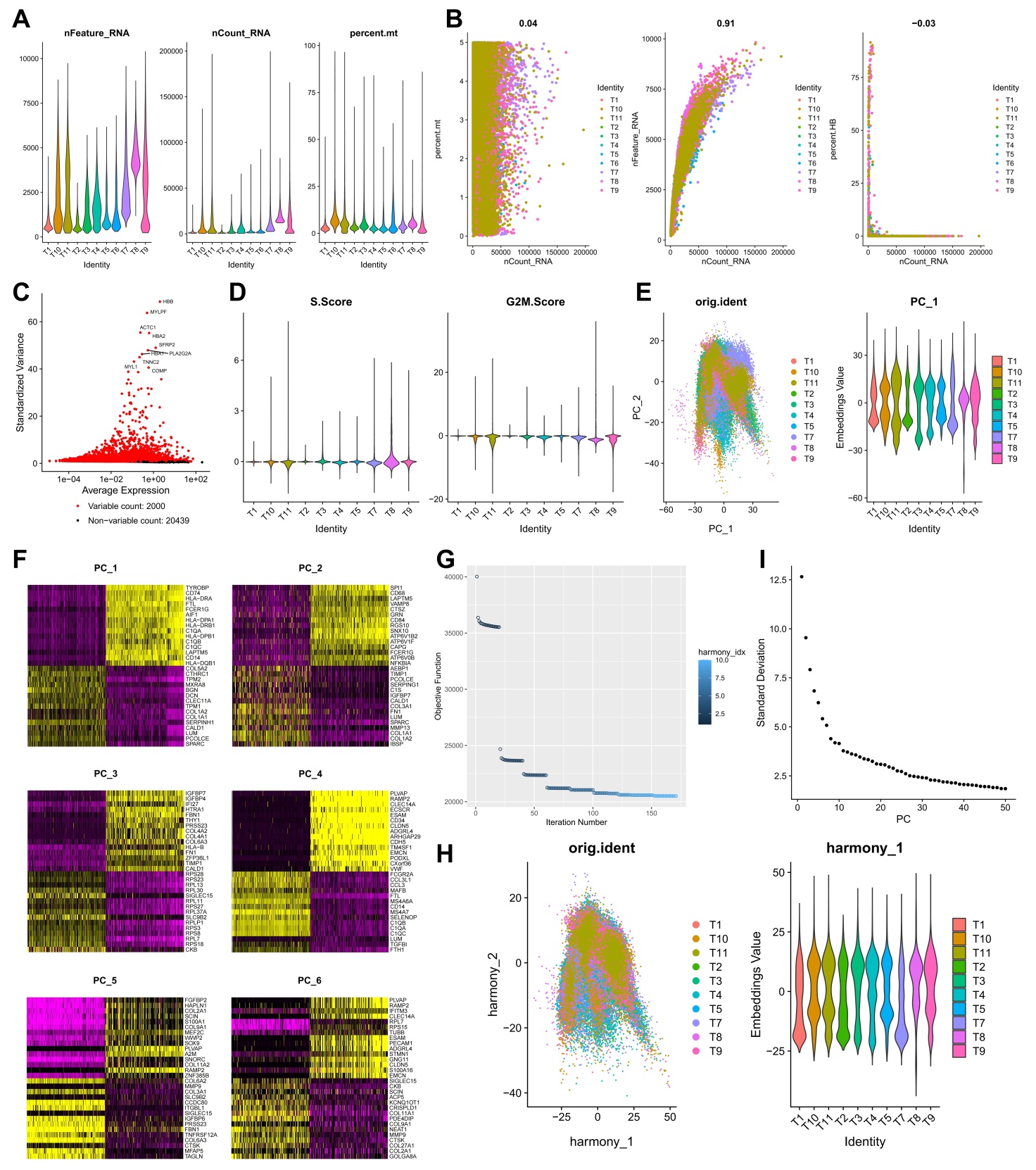
**

**Figure S5. Quality Control and PCA Dimension Reduction of scRNA-seq Data.**

Note: (A) Violin plots displaying the number of genes per cell (nFeature_RNA), the number of mRNA molecules per cell (nCount_RNA), and the percentage of mitochondrial genes (percent.mt) in scRNA-seq data (N=11); (B) Scatter plots showing the correlation between filtered data - nCount_RNA and percent.mt, nCount_RNA and nFeature_RNA, nCount_RNA and percent.HB (N=11); (C) Variance analysis for selecting highly variable genes, with red indicating the top 2000 highly variable genes, black indicating genes with low variability, and the top 10 gene names in highly variable genes labeled (N=11); (D) Cell cycle states of each cell in scRNA-seq data, where S.Score represents S phase, and G2M.Score represents G2M phase (N=11); (E) Distribution of cells in PC_1 and PC_2 analyzed by PCA, with each point representing a single cell (N=11); (F) Heatmap of the top 20 genes most associated with PC_1 - PC_6 in the PCA, where yellow indicates upregulated expression and purple indicates downregulated expression (N=11); (G) Batch correction process using Harmony, with the x-axis representing the number of interactions; (H) Distribution of cells in PC_1 and PC_2 after batch correction by Harmony, with each point representing a single cell; (I) Distribution of standard deviations of PCs, where important PCs have larger standard deviations (N=11).

**
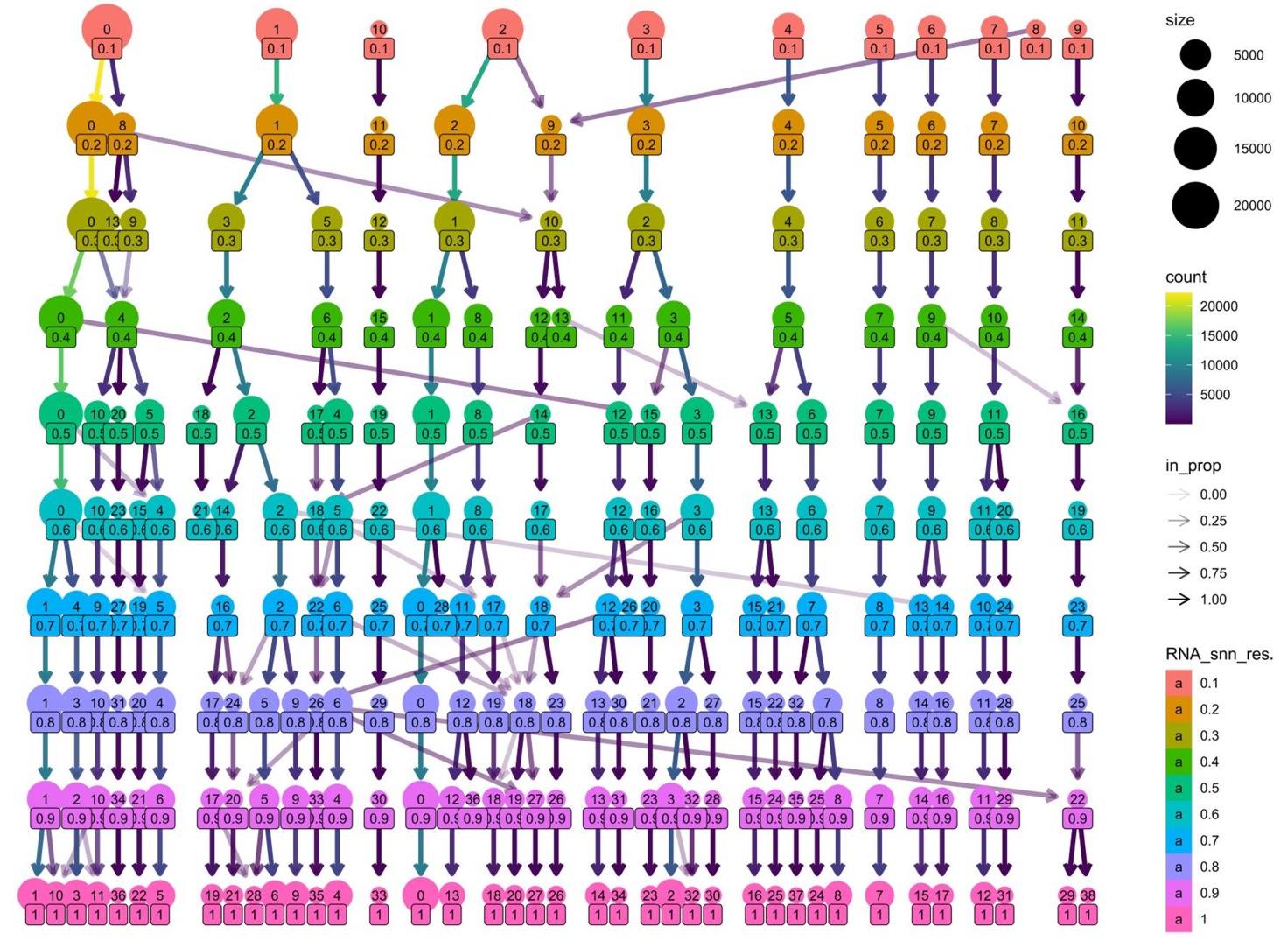
**

**Figure S6. UMAP Clustering Dendrogram of scRNA-seq Data.**

**
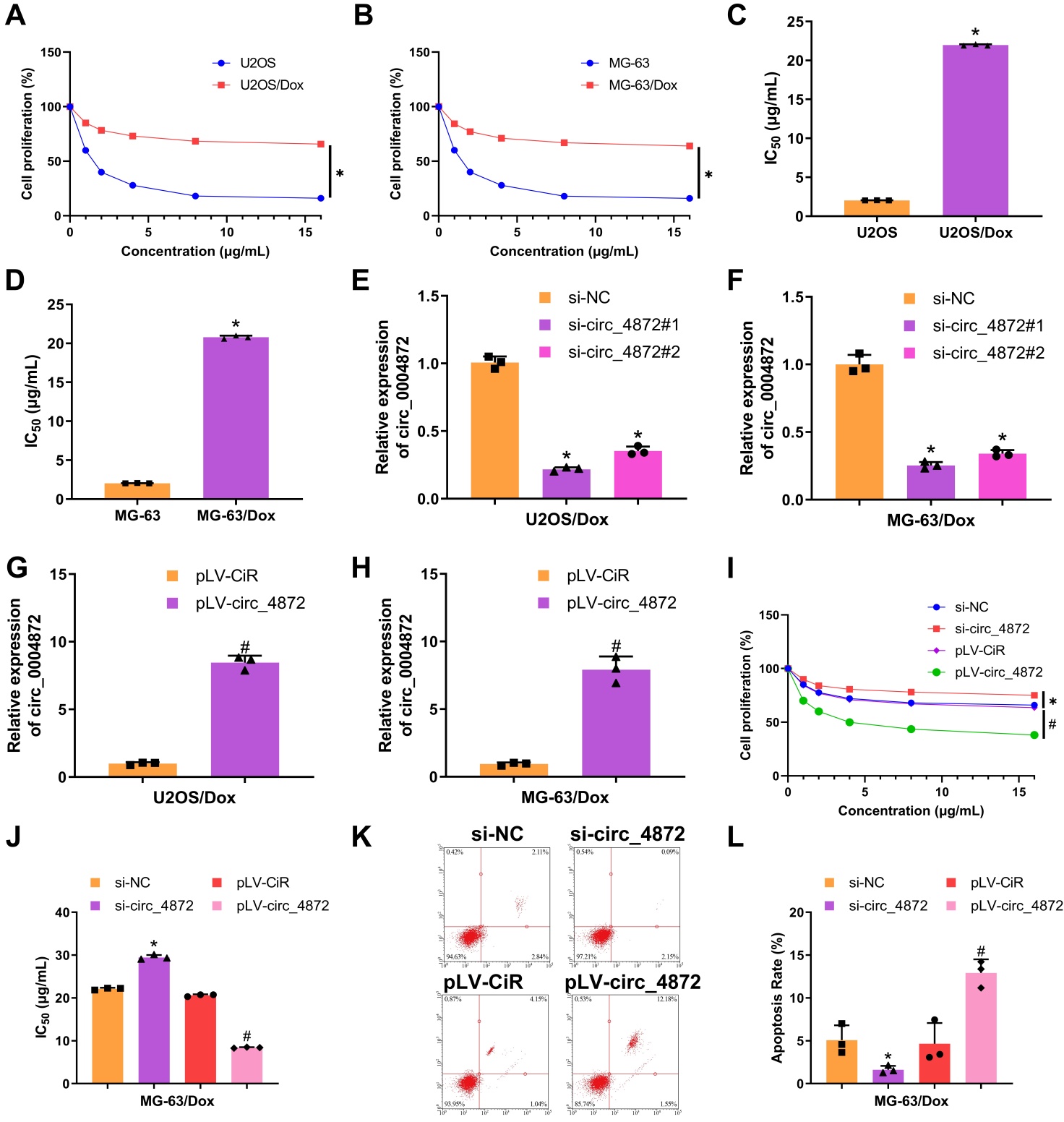
**

**Figure S7. hsa_circ_0004872 Impact on Chemoresistance in OS Cells.**

Note: (A-B) CCK-8 assay to evaluate cell viability in MG-63 and U2OS cells; (C-D) determination of IC_50_ values in MG-63 and U2OS cells; (E-H) efficiency of knockdown or overexpression of hsa_circ_0004872 by RT-qPCR; (I) CCK-8 assay measuring cell viability in MG-63/Dox cells; (J) determination of IC_50_ value in MG-63/Dox cells; (K-L) flow cytometry analysis of apoptosis in different groups of MG-63/Dox cells. Cell experiments were conducted at least three times, where * indicates significance compared to U2OS, MG-63, si-NC groups at *p* < 0.05, and # indicates significance compared to the PLV-CIR group at *p* < 0.05.

**
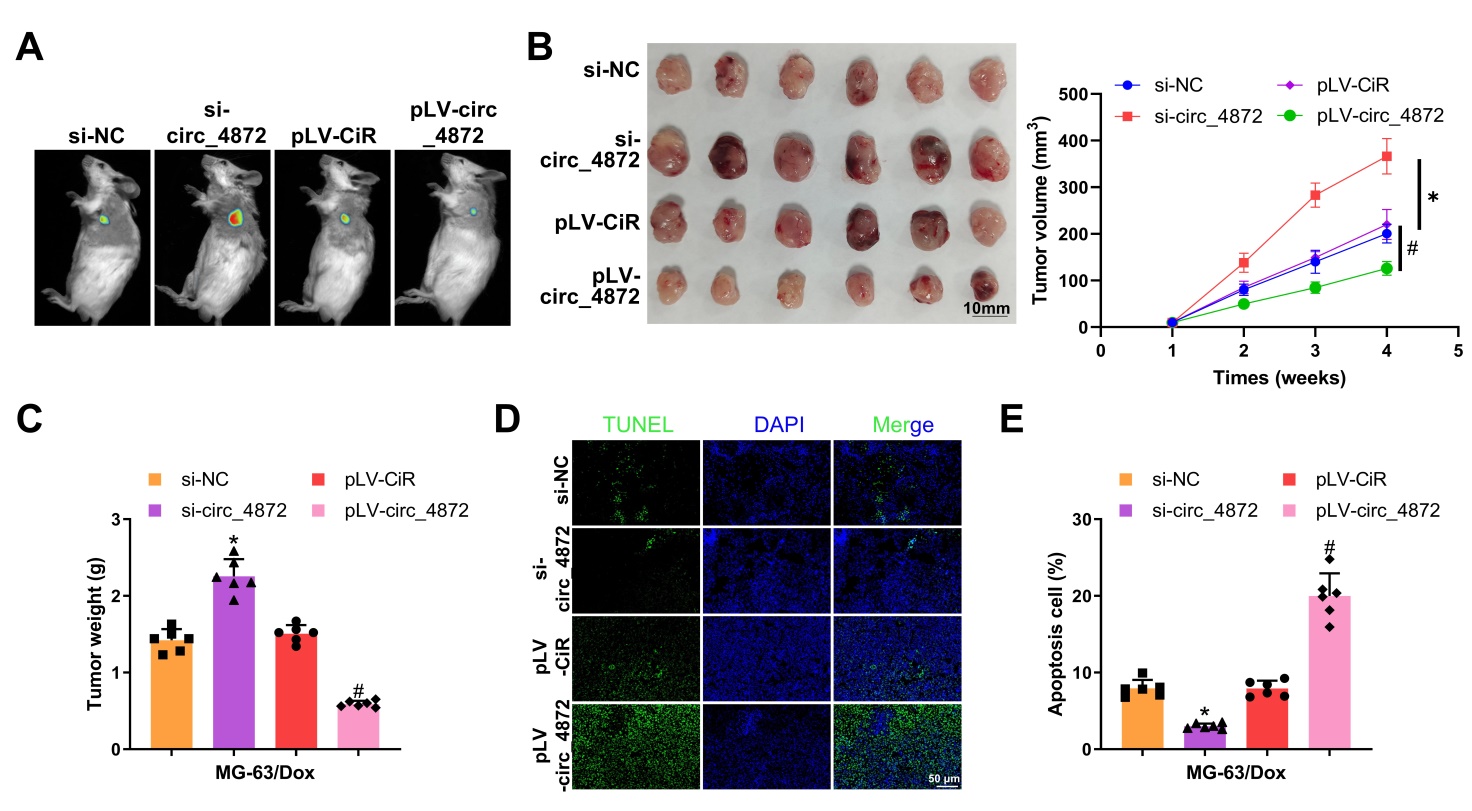
**

**Figure S8. Impact of hsa_circ_0004872 on Tumor Formation *In Vivo* in MG-63/Dox Cells.**

Note: (A) Tumor growth at different time points monitored by bioluminescence intensity, with one representative example shown for each group; (B) Morphology and growth status of tumor tissues in each group of mice; (C) Weight of tumor tissues in each group of mice; (D-E) Detection of cell apoptosis in tumor tissues of each group of mice using TUNEL assay, where TUNEL green marks apoptotic cells, and DAPI blue marks cell nuclei (scale bar: 50 μm). * indicates *p* < 0.05 compared to the si-NC group, # indicates *p* < 0.05 compared to the PLV-CIR group, with 6 mice in each group.

**
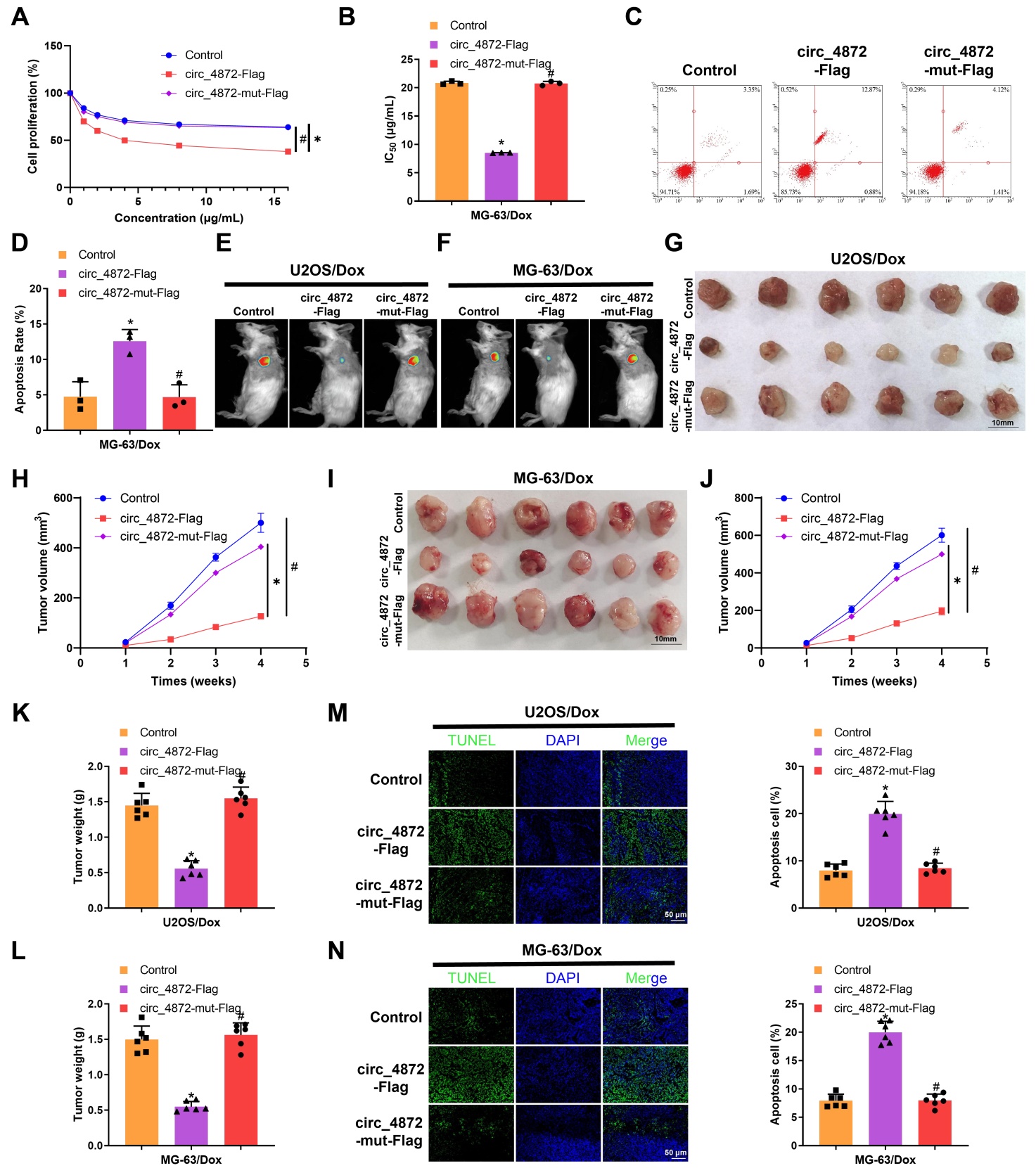
**

**Figure S9. Small Peptide Encoded by hsa_circ_0004872 Influences *In Vivo* Tumorigenesis of OS Cells.**

Note: (A) Evaluation of MG-63/Dox cell viability by CCK-8 assay; (B) IC_50_ values of MG-63/Dox cells; (C-D) Flow cytometry analysis of apoptosis in MG-63/Dox cells in different groups; (E-F) Tumor growth monitored by bioluminescence intensity at various time points, with each group represented by a single example; (G-J) Morphology of tumor tissues in different groups of mice (G, I), as well as tumor growth status (H, J); (K-L) Tumor tissue weight in each group of mice; (M-N) Assessment of cell apoptosis in tumor tissues of each group of mice using TUNEL assay, where TUNEL green marks apoptotic cells and DAPI blue marks cell nuclei (scale bar: 50 μm). * indicates *p* < 0.05 compared to the Control group, # indicates *p* < 0.05 compared to the circ_4872-Flag group. Cell experiments were performed in triplicates, with 6 mice in each group.

**
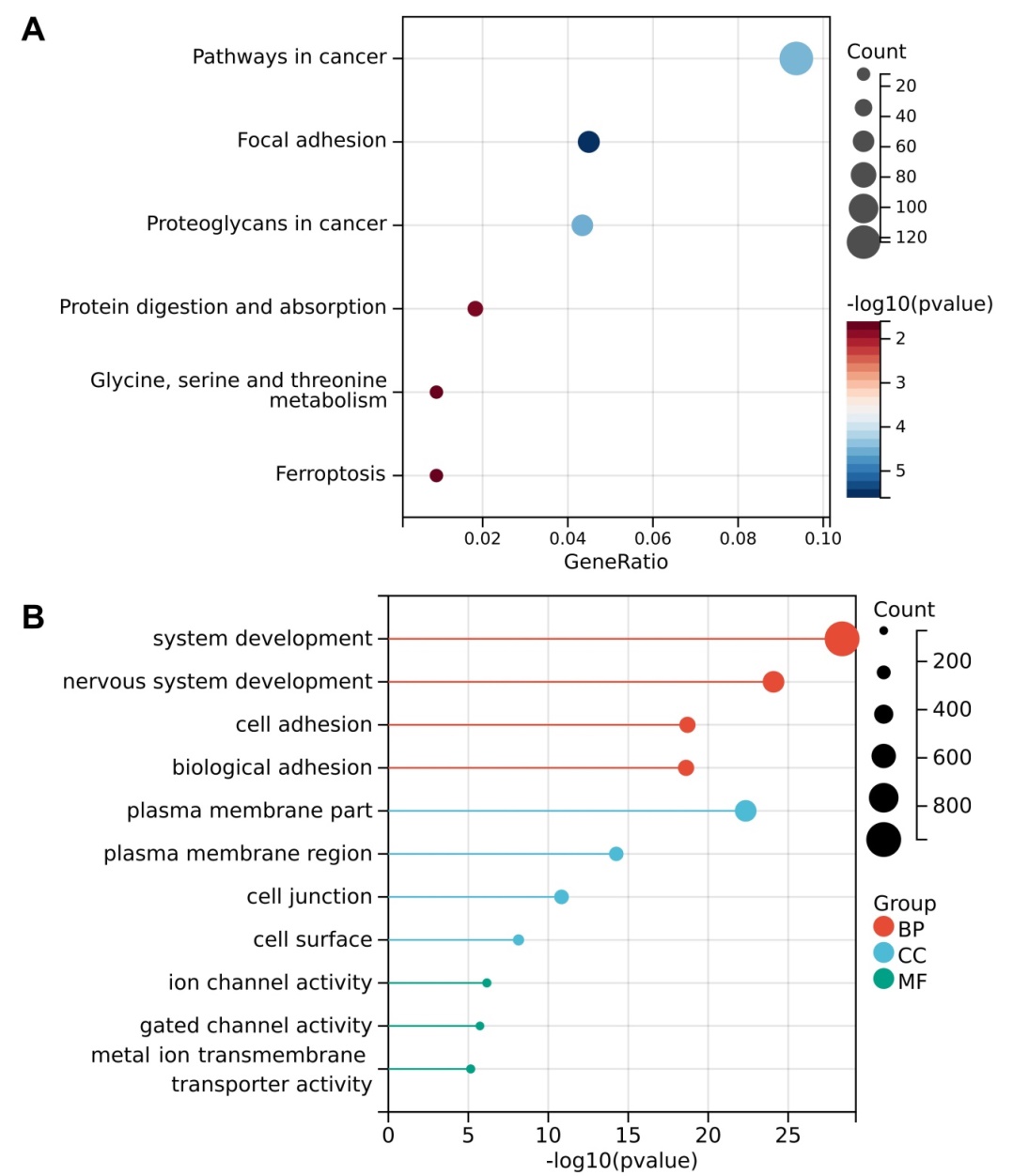
**

**Figure S10. Pathway Enrichment Analysis of DEGs.**

Note: (A) Bar chart showing the GO enrichment of DEGs in U2OS/Dox cell samples, where red, blue, and green correspond to BP, CC, and MF categories respectively; (B) KEGG pathway circular diagram depicting DEGs in U2OS/Dox cell samples.

**
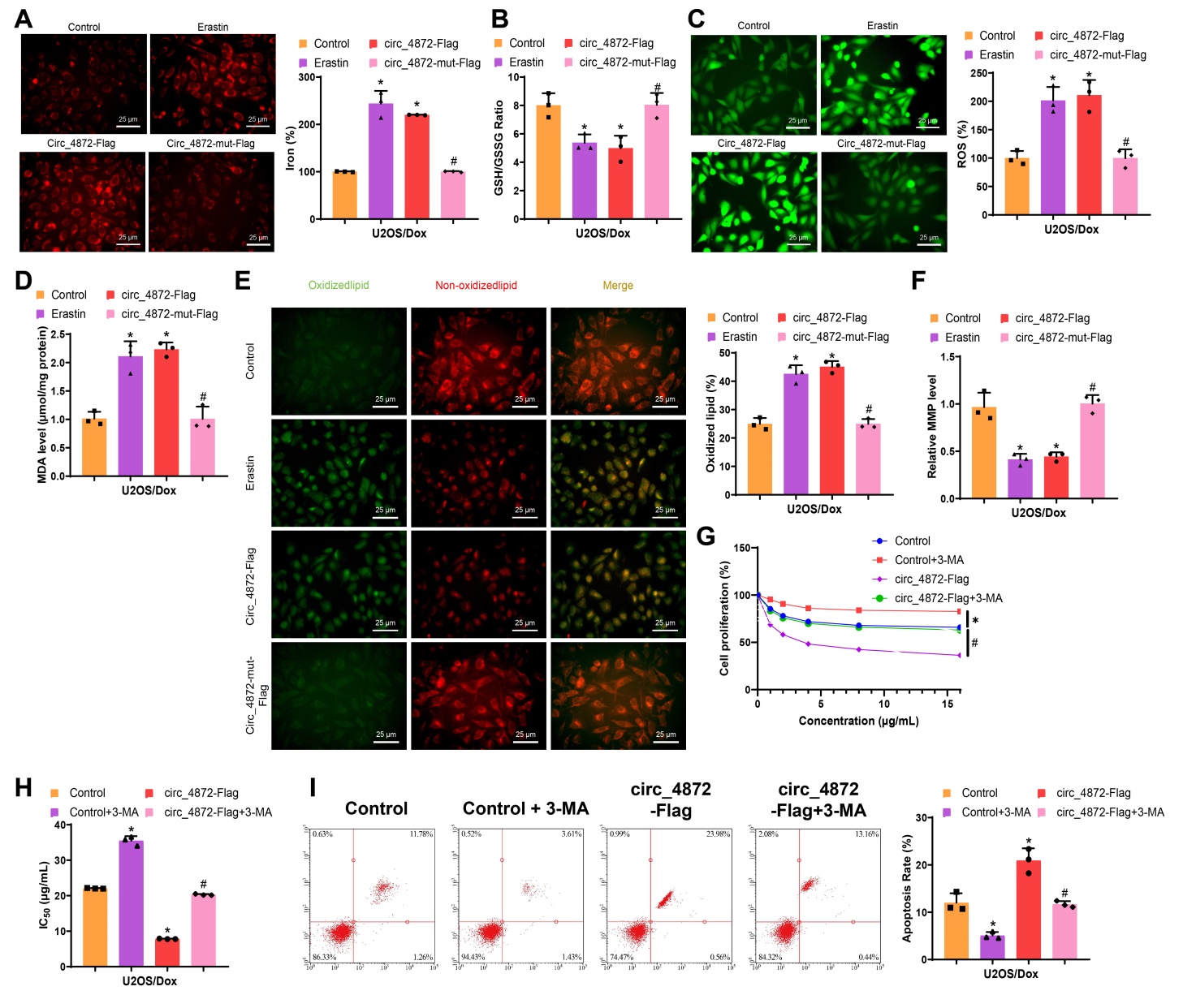
**

**Figure S11. Impact of Small Molecular Peptides Encoded by hsa_circ_0004872 on Ferroptosis in U2OS/Dox Cells.**

Note: (A) Assessment of iron levels in U2OS/Dox cells using FerroOrange (Scale bar=25 μm); (B) GSH/GSSG ratio in U2OS/Dox cells post different treatments; (C) Visualization of ROS generation in U2OS/Dox cells using DCFH-DA under confocal laser scanning microscopy and statistical analysis by flow cytometry, Scale bar=25 μm; (D) Detection of MDA content in each group; (E) Representative confocal images of C11-BODIPY 581/591 stained U2OS/Dox cells. Red indicates unoxidized lipids, green represents oxidized lipids (Scale bar=25 μm); (F) JC-1 assessment of mitochondrial membrane potential (MMP) in U2OS/Dox cell groups; (G) Assessment of cell viability in different U2OS/Dox cell groups using CCK-8 assay; (H) IC_50_ values of U2OS/Dox cells in each group; (I) Flow cytometry analysis of apoptosis in U2OS/Dox cell groups. * indicates *p* < 0.05 compared to the Control group, # indicates *p* < 0.05 compared to the circ_4872-Flag group, cell experiments were replicated at least three times.

**
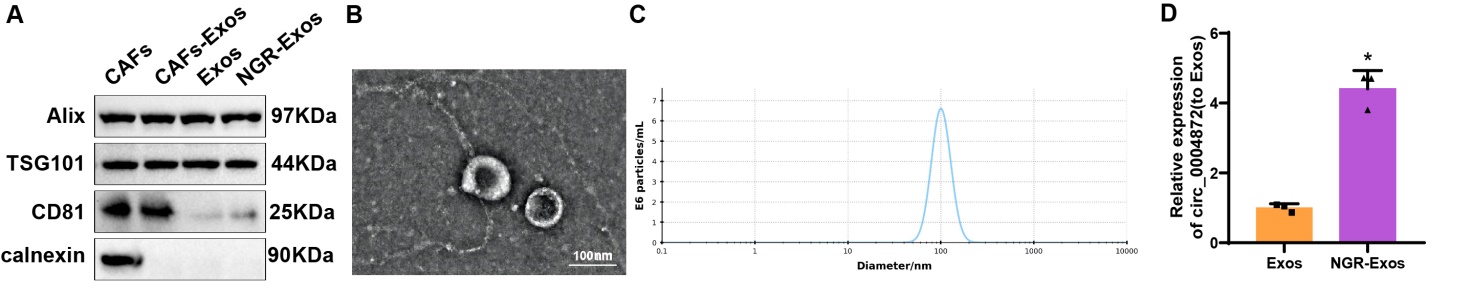
**

**Figure S12. Identification of CAFs Exos.**

Note: (A) Western blot analysis of the protein expression of Alix, TSG101, and CD81 in EVs; (B) Morphological observations of CAFs-EVs via TEM, where white arrows indicate classic discoidal vesicular structures (scale bar: 100 nm); (C) Size distribution analysis of CAFs-EVs by nanoparticle tracking; (D) RT-qPCR detection of the loading capacity of circ_0004872-109aa in Exos and NGR-Exos. Cell experiments were replicated at least three times.

**
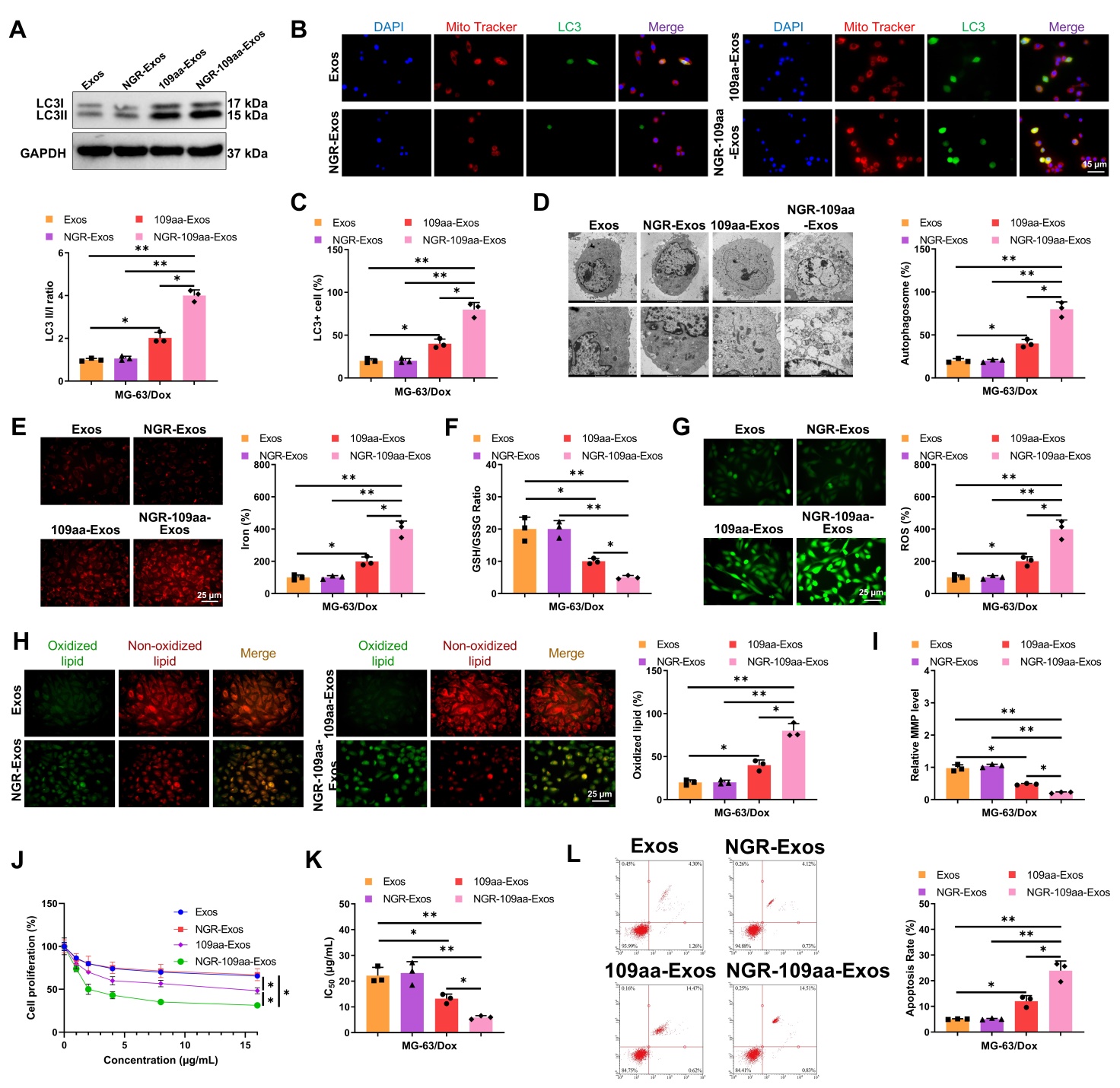
**

**Figure S13. Impact of NGR-109aa-Exos on Autophagy-Dependent Ferroptosis in MG-63/Dox Cells.**Note: (A) Expression levels of mitochondrial autophagy-related proteins LC3I/II in MG-63/Dox cells were detected by Western blot; (B-C) Co-localization results of mitochondria and LC3 in M2 macrophages in each group were observed under confocal laser microscopy (scale bar: 15 μm); (D) Mitochondrial morphology of cells was examined by TEM; (E) Intracellular iron levels in MG-63/Dox cells were identified using FerroOrange (Scale bar = 25 μm); (F) Ratios of GSH/GSSG in MG-63/Dox cells after different treatments; (G) Visualization of ROS production in MG-63/Dox cells using DCFH-DA under confocal laser scanning microscopy, followed by statistical analysis using flow cytometry, with a scale bar of 25 μm; (H) Representative confocal images of C11-BODIPY 581/591-stained MG-63/Dox cells, where red indicates unoxidized lipids and green represents oxidized lipids (Scale bar = 25 μm); (I) Measurement of mitochondrial membrane potential (MMP) in MG-63/Dox cells using JC-1 dye; (J) Assessment of cell viability of MG-63/Dox cells using CCK8 assay; (K) Determination of IC_50_ values for MG-63/Dox cells; (L) Detection of apoptosis in MG-63/Dox cells by flow cytometry. **p* < 0.05, ***p* < 0.01, cell experiments were conducted at least three times.

**
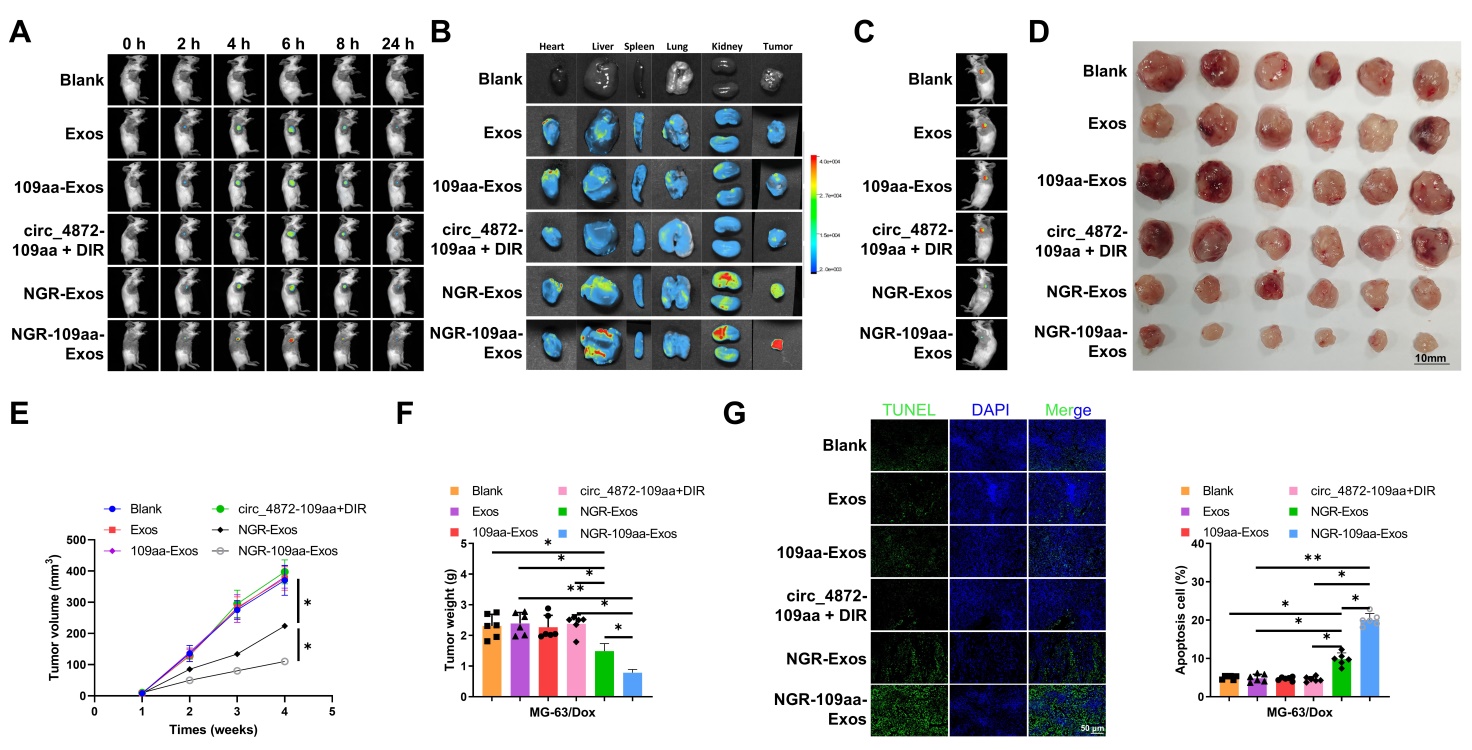
**

**Figure S14. NGR-109aa-Exos Influence Tumor Formation of MG-63/Dox Cells *In Vivo*.**

Note: (A) NIR fluorescence imaging to observe the *in vivo* fluorescence distribution in tumor-bearing nude mice; (B) NIR fluorescence imaging to observe the fluorescence distribution in major organs and tumor tissues of nude mice in each group; (C) Monitoring of tumor growth at different time points through bioluminescence intensity, with one representative example shown for each group; (D) Morphology of tumor tissues in each group of mice; (E) Tumor growth status in each group of mice; (F) Tumor tissue weight in each group of mice; (G) TUNEL assay to detect cell apoptosis in tumor tissues of each group of mice, with TUNEL staining apoptotic cells in green and DAPI staining cell nuclei in blue (scale bar: 50 μm). **p* < 0.05, n=6 mice per group.

**
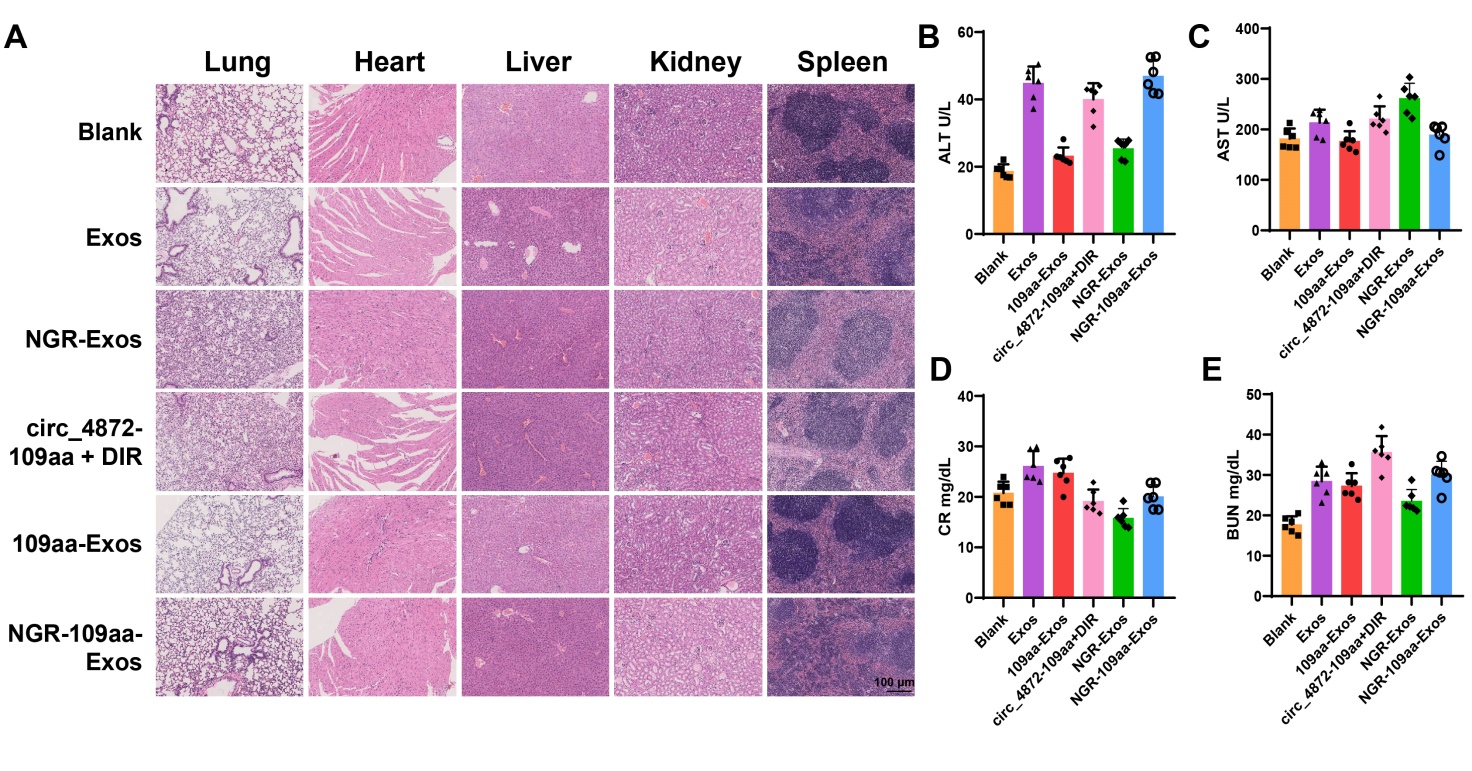
**

**Figure S15. Histological Images of Major Organs in Mice Stained with H&E.**

Note: (A) H&E staining images of lung, heart, liver, kidney, and spleen sections from each group of mice; scale bar: 100 μm; six mice per group; (B-E) Biochemical analysis results of alanine aminotransferase (ALT), aspartate aminotransferase (AST), creatinine (CRE), and blood urea nitrogen (BUN) levels in the blood of mice from different groups; six mice per group.

**Table S1. Cell Types and Marker Genes.**

| **Cell Type** | **Marker genes** |
| --- | --- |
| B Cell | SSPN, BHLHE41, IGHA1 |
| Cancer-Associated Fibroblast (CAF) | IGFBP5, TAGLN, MEG3 |
| Dendritic Cell | TNFSF10, IFIT3, IFIT2 |
| Endothelial Cell | RAMP2, GNG11, PLVAP |
| Macrophage | APOC1, APOE, C1QB |
| Natural Killer T (NKT) Cell | RPL37A, RPL37, RPL36 |
| Osteoclast | CTSK, CD40LG |
| Osteoblast | THY1, ENG |
| Progenitor Cell | LUM, PCOLCE, TOP2A |
| T Cell | IL32, CCL5, CD3D |

**Table S2. siRNA Sequences.**

| **siRNAs** | **Sequence** |
| --- | --- |
| si-circ_4872#1 | TTCCAAGCTCTGCTTATGAT |
| si-circ_4872#2 | GTTGAATTCCAAGCTCTGCTT |
| si-NC | UAGACUGAGUUAACUUAA |

**Table S3. RT-qPCR Primer Sequences.**

| **Gene** | **Primer Sequence** |
| --- | --- |
| GAPDH (human) | F: 5'-CGGATTTGGTCGTATTGGGC-3' |
|  | R: 5'-TTGACGGTGCCATGGAATTTG-3' |
| Hsa_circ_0004872 (Divergent primer,181bp) | F: 5'-GTTGCAGATCCAGACCATGA-3' |
|  | R: 5'-CAGGGTTCTCTGGCAGTAGG-3' |
| Hsa_circ_0004872 (Convergent primer,180bp) | F: 5'-ACAACACCTCAGCAATGACCA-3' |
|  | R: 5'-TGGTCTGGATCTGCAACACG-3' |

Note: F: Forward. R: Reverse.

**Table S4. Details of the First Antibody Product.**

| **Name** | **Cat.** | **Dilution ratio** | **Manufacturer** | **Country** | **MW (kDa)** |
| --- | --- | --- | --- | --- | --- |
| GAPDH | ab9485 | 1: 2500 | Abcam | UK | 37 |
| LC3II/I | PA5-22731 | 1: 1000 | Invitrogen | USA | 15/17 |
| CD63 | MA5-35208 | 1: 1000 | Invitrogen | USA | 40 |
| 6x-His Tag | MA1-21315 | 1: 1000 | Invitrogen | USA | / |
| Flag (DDDDK) | ab205606 | 1: 500 | Abcam | UK | / |
